# Effect of Cancer Pain Guideline Implementation on Pain Outcomes Among Adult Outpatients With Cancer-Related Pain: A Stepped Wedge Cluster Randomized Trial

**DOI:** 10.1001/jamanetworkopen.2022.0060

**Published:** 2022-02-21

**Authors:** Melanie R. Lovell, Jane L. Phillips, Tim Luckett, Lawrence Lam, Frances M. Boyle, Patricia M. Davidson, Seong L. Cheah, Nicola McCaffrey, David C. Currow, Tim Shaw, Annmarie Hosie, Bogda Koczwara, Stephen Clarke, Jessica Lee, Martin R. Stockler, Caitlin Sheehan, Odette Spruijt, Katherine Allsopp, Alexandra Clinch, Katherine Clark, Alison Read, Meera Agar

**Affiliations:** 1 Palliative Care Department, HammondCare, Greenwich, Australia; 2 Northern Clinical School, Sydney Medical School, Sydney, Australia; 3 IMPACCT Centre—Improving Palliative, Aged and Chronic Care through Clinical Research and Translation University of Technology Sydney, Sydney, Australia; 4 Patricia Ritchie Centre for Cancer Care and Research, University of Sydney, Sydney, Australia; 5 Johns Hopkins University School of Nursing, Baltimore, Maryland; 6 Deakin University, Geelong, Deakin Health Economics, Institute for Health Transformation, School of Health and Social Development, Victoria, Australia; 7 Faculty of Science, Medicine and Health, University of Wollongong, Wollongong, Australia; 8 University of Sydney, Sydney, Australia; 9 The University of Notre Dame Australia, School of Nursing Sydney and St Vincent’s Health Network Sydney, Sydney, Australia; 10 Flinders Medical Centre and Flinders University, Adelaide, Australia; 11 Royal North Shore Hospital, St Leonards, Australia; 12 Concord Centre for Palliative Care, Concord Repatriation General Hospital, Sydney, NSW, Australia; 13 University of Sydney, Concord Clinical School, Australia; 14 Palliative Care Department, South East Sydney Local Health District, Southern Sector, Sydney Australia; 15 Palliative Care Department, Western Health, University of Melbourne, Melbourne, Australia; 16 Palliative Care Department, Westmead Hospital, Westmead, Sydney, Australia; 17 Department of Palliative Care, Peter Macallum Cancer Centre, Melbourne, Australia; 18 University of Wollongong, Wollongong, Australia

## Abstract

**Importance:**

An evidence-practice gap exists for cancer pain management, and cancer pain remains prevalent and disabling.

**Objectives:**

To evaluate the capacity of 3 cancer pain guideline implementation strategies to improve pain-related outcomes for patients attending oncology and palliative care outpatient services.

**Design, Setting, and Participants:**

A pragmatic, stepped wedge, cluster-randomized, nonblinded, clinical trial was conducted between 2014 and 2019. The clusters were cancer centers in Australia providing oncology and palliative care outpatient clinics. Participants included a consecutive cohort of adult outpatients with advanced cancer and a worst pain severity score of 2 or more out of 10 on a numeric rating scale (NRS). Data were collected between August 2015 and May 2019. Data were analyzed July to October 2019 and reanalyzed November to December 2021.

**Interventions:**

Guideline implementation strategies at the cluster, health professional, and patient levels introduced with the support of a clinical champion.

**Main Outcomes and Measures:**

The primary measure of effect was the percentage of participants initially screened as having moderate to severe worst pain (NRS ≥ 5) who experienced a clinically important improvement of 30% or more 1 week later. Secondary outcomes included mean average pain, patient empowerment, fidelity to the intervention, and quality of life and were measured in all participants with a pain score of 2 or more 10 at weeks 1, 2, and 4.

**Results:**

Of 8099 patients screened at 6 clusters, 1564 were eligible, and 359 were recruited during the control phase (mean [SD] age, 64.2 [12.1] years; 196 men [55%]) and 329 during the intervention phase (mean [SD] age, 63.6 [12.7] years; 155 men [47%]), with no significant differences between phases on baseline measures. The mean (SD) baseline worst pain scores were 5.0 (2.6) and 4.9 (2.6) for control and intervention phases, respectively. The mean (SD) baseline average pain scores were 3.5 (2.1) for both groups. For the primary outcome, the proportions of participants with a 30% or greater reduction in a pain score of 5 or more of 10 at baseline were similar in the control and intervention phases (31 of 280 participants [11.9%] vs 30 of 264 participants [11.8%]; OR, 1.12; 95% CI, 0.79-1.60; *P* = .51). No significant differences were found in secondary outcomes between phases. Fidelity to the intervention was low.

**Conclusions and Relevance:**

A suite of implementation strategies was insufficient to improve pain-related outcomes for outpatients with cancer-related pain. Further evaluation is needed to determine the required clinical resources needed to enable wide-scale uptake of the fundamental elements of cancer pain care. Ongoing quality improvement activities should be supported to improve sustainability.

## Key Points

**Question:**

Can guideline implementation strategies improve pain outcomes in adult outpatients with cancer-related pain?

**Findings:**

In this cluster-randomized trial of 544 patients, a suite of implementation strategies comprising audit and feedback, clinician-spaced education, and a patient self-management resource was evaluated. There was no statistical difference in pain-related outcomes, and fidelity, which was measured by uptake of the 3 implementation strategies, was lower than expected.

**Meaning:**

These findings suggest that cancer pain screening and subsequent management will need to be highly valued, supported by adequate resources embedded within the clinical workflow, and supported by electronic data systems to be successful.

## Teaser

This stepped wedge, cluster randomized trial evaluated the capacity of 3 cancer pain guideline implementation strategies to improve pain-related outcomes for patients attending oncology and palliative care outpatient services.

## Introduction

Cancer pain is a prevalent, disabling, and distressing symptom.^1^ The prevalence of pain of moderate or severe intensity is greater than 50% in patients with advanced cancer and is undertreated in around one-third of individuals.^1^ Beyond personal suffering for individuals and their families, untreated pain is associated with higher health care use.^2,3,4^ Clinical practice guidelines provide evidence-based recommendations for pain management; however, adoption and adherence are suboptimal.^5^ A greater focus on strategies to foster implementation is needed.^6^ Barriers to guideline implementation exist at patient, health professional, and health service levels suggesting the need for multifaceted strategies.^7,8,9^

Effective cancer pain assessment and management requires patient self-report using patient-reported outcome measures (PROMs) and adherence to management plans; health professionals with the commitment, knowledge, and skills to manage pain; and health services to provide a method of screening to identify and monitor symptoms.^6,10^ Screening for pain in cancer outpatients has been shown to improve quality of care and, more recently, pain-related outcomes.^11^ However, as with guidelines, clinicians are unlikely to use symptom screening to inform pain management unless they are motivated and supported by their health systems either locally, regionally, or nationally.^12^ Other strategies known to improve pain outcomes include patient education^8,13^ and health professional education.^14,15^ Audit and feedback have proven effects on professional practice and health care outcomes.^16^ Implementation of cancer pain assessment and management improvement strategies has been shown to be effective in inpatient settings.^17^ However, implementation has been met with variable success in the ambulatory care setting.^18,19^

This study’s primary aim was to measure if the intervention resulted in a 30% improvement in pain score in adults attending oncology or palliative care outpatient clinics with a pain Numeric Rating Scale (NRS) score of 5 or more. Four secondary aims were included to evaluate the capacity of 3 guideline implementation strategies compared with standard care in people screened for cancer pain alone. First, we evaluated the implementation strategies’ ability to reduce the mean worst pain and mean average pain severity throughout the past 24 hours across patients screened as having clinically relevant (ie, NRS ≥ 2) or moderate to severe (ie, NRS ≥ 5) worst pain from the time of screening to 1, 2, and 4 weeks later by an SD of 0.5.^20^ Second, we evaluated their ability to find a between-phase difference in patient empowerment as measured by 0.5-SD difference on the Health Education Impact Questionnaire (heiQ),^21^ mean patient quality of life (QOL), as measured by a difference of 0.5 SD on the European Organisation for Research and Treatment of Cancer Palliative Care (EORTC QLQ C15-PAL),^22^ and difference in the experiences of unpaid carers of participating patients, as measured by a 0.5-SD difference on the Carer Experience Scale (CES)^23^ at 2 and 4 weeks after screening. Third, we evaluated the implementation strategies’ capacity to assess the cost-effectiveness of the 3 guideline implementation strategies based on incremental cost per additional responder on the primary outcome. Fourth, we assessed their fidelity to the intervention. The current article reports results relating to the primary aim and the first and third of the secondary aims.

## Methods

This stepped-wedge, cluster-randomized trial was reported according to the Consolidated Standards of Reporting Trials Extension (CONSORT Extension) reporting guideline.^24^ Ethical approval was granted by the human research ethics committee of the Southwestern Sydney Local Health District. Following commencement of the trial, an approved amendment allowed for patients to provide verbal consent rather than written consent to ensure timely collection of data at week 1. The methods are reported in Luckett et al^25^ and summarized below. The trial protocol can be found in Supplement 1.

### Study Design and Participants

#### Design

A stepped wedge, cluster-randomized trial approach was taken in which clusters were randomized to commence the intervention at different times following an initial control period in which outcomes were measured for usual care. A training phase enabled the transition from control to intervention, during which recruitment and measurement were placed on hold.

#### Participants and Setting

The clusters were cancer centers, which provided medical and radiation oncology, and palliative care clinics. Purposive sampling was used to ensure that a range of metropolitan and regional centers from various states or territories across Australia were represented. Eligible participants were adults with advanced cancer and a worst pain severity of 2 or more out of 10 on an NRS, who were able to complete the NRS in 1 of the 6 most frequent languages spoken in Australia (ie, English, Italian, Greek, Arabic, Mandarin, or Vietnamese) who did not opt out. Caregivers were eligible if they were identified by a patient, provided consent, and had sufficient English to give informed consent and complete the measures. Staff were eligible if they were employed permanently, either full- or part-time, and provided care or administrative support to patients at a participating cancer center.

#### Recruitment

Our study design enabled the intervention to target practice change at the center level without risking contamination between phases and control for practice variation between centers. Centers were randomized to transition one at a time. Each patient was recruited to participate in either the control or intervention phase, but not both, thus, samples differed between phases and consent was obtained. The time between each step was not predetermined by dates but rather by the readiness of centers to commence and the speed they recruited to the target sample size. A sustainability phase was also included in the design wherein medical records of patients screened as having severe pain (≥7 NRS) were audited at 3-month intervals from the end of the intervention phase to the end of the study period to assess whether adherence to guidelines for pain screening, assessment, and management were continued.

### Randomization

The order in which each center moved from control to intervention phase was randomly allocated by a computer algorithm performed by the study statistician. Allocation of clusters could not be concealed from clinicians and managers. Blinding for center staff and the project team collecting data was not possible. However, information for patients provided only general information about the aims of the study, not the specifics of the design and intervention. Patients were allocated based on whether the center they attended was in the control or intervention phase at the time they were first screened as having worst pain rated 2 or more on the NRS.

### Treatment Allocation

#### Control

A paper-based screening system was implemented at each center whereby all patients were requested to complete an NRS for worst pain and average pain during the past 24 hours on each visit. Pain screening was administered to patients in the waiting room either by administrative personnel or nursing staff, depending on the center. Each center provided usual care, as per local practice. No attempt was made to encourage or discourage pain screening data being used to inform patient consultations.

#### Training and Intervention

During the training phase, center clinicians were given an overview of the guidelines and implementation resources by a local clinical champion with support from the project team. A system was established at each center to enable screening results to be communicated to the physician to inform the consultation. Guideline implementation strategies developed using a conceptual framework (Michie Behavior Change Wheel)^26^ included: (1) audit of adherence to 6 key guideline recommendations and feedback delivered in 1 to 2 cycles; (2) health professional education using email-administered spaced education via the Qstream platform;^27^ and (3) an education booklet and a patient self-management resource. These strategies have a positive impact when implementing guidelines either in cancer pain or other clinical contexts but have never been tested in combination.

### Outcomes

Patient-reported outcome measures were obtained by researchers contacting participants by phone who scored 2 or more on either NRS at the time of screening. If the participant could not be reached after 3 attempts on 3 consecutive days, their data were categorized as missing for that time point.

The primary study outcome was the percentage of patients at the cluster level screened with a score of 5 or more out of 10 on a worst pain NRS with a pain reduction of 30% from the initial NRS at week 1. Thirty percent was selected based on the US Food and Drug Administration recommendations, which indicate that measurement of minimal clinically important difference should take account of the baseline score.^28^ Pain rated as 5 on the NRS was selected as an established threshold for moderate pain.^29^ Secondary outcomes were collected via telephone at weeks 1, 2, and 4 after screening and consisted of worst pain and average pain NRS, patient empowerment heiQ,^21^ carer experience with the CES^23^, and patient QOL using the EORTC QLQ-C15-PAL.^22^ Data collection deviated from the protocol by collecting only the following domains of the heiQ after the whole measure was found too burdensome for patients: social integration and support, health service navigation, constructive attitudes and approaches, and skill and technique acquisition.

Fidelity was assessed for each part of the intervention. The number of patients receiving written pain education materials was assessed by asking the patient over the telephone at week 1 if they had received them. This was implemented after the first site completed and hence, we have data for 5 sites only. The clinicians’ completion of the online spaced education module was measured via the administering platform of QStream platform.^14^ The number of audit and feedback rounds was counted.

### Statistical Analysis

We used a carefully designed computer simulation, allowing for 20% dropout by both centers and patients, to estimate statistical power. Drawing on data from 1612 consecutive patients,^30^ outcome data were generated to mimic the pain scores we expected to see in the presenting population. Data were analyzed using Stata version 17 BE (StataCorp). Sample size required was 82 at 6 sites totaling 492. The sample size calculation was based on the statistical approach for analyses being the Generalized Linear Mixed Model with a 2-tailed test. Pearson χ^2^ tests or the Student *t* test were used to compare baseline patients’ characteristics between control and intervention phases. To cater for the cluster effect in the primary outcome measure, the generalized linear mixed model with a binary outcome was applied to examine the differences in proportions of pain score reduction by 30% between phases with adjustment for possible confounding variables, such as age, gender, cancer type, Qstream, information pack, audit cycles, and the time spent in each phase. A similar analytical approach was adopted to analyze all secondary outcome measures with adaptation for continuous variables. For testing of the hypothesis, a 2-tailed error rate of 5% was used.

A statistically significant level of *P* < .05 was used for all tests. Data were collected between August 2015 and May 2019. Data were analyzed July 2019 to October 2019 and data were reanalyzed in November and December 2021.

## Results

### Participants

**Figure 1.**
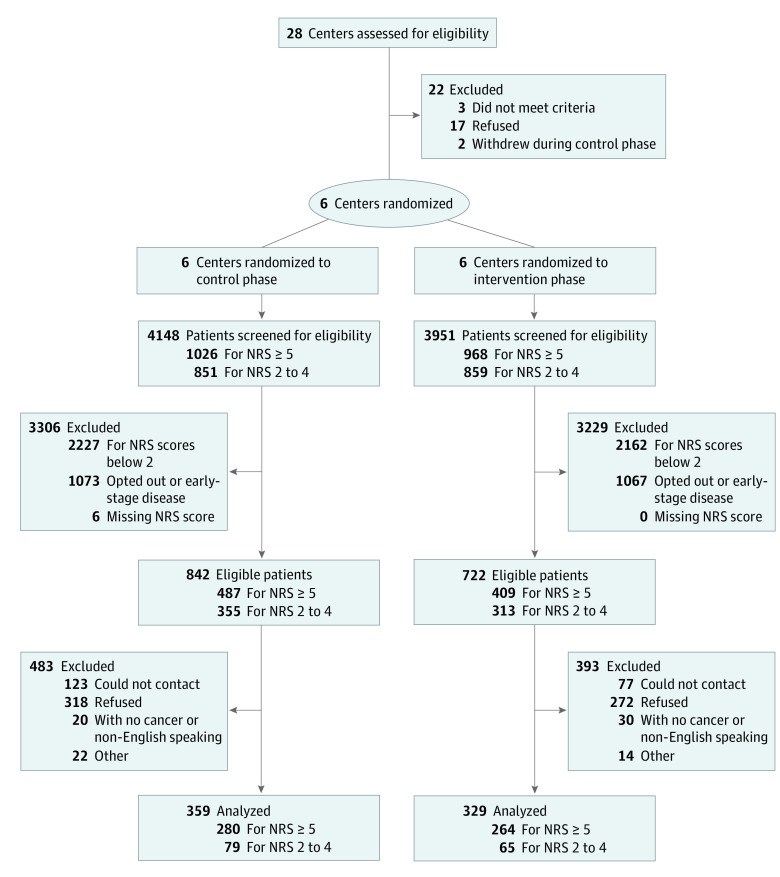
Flow Diagram

This study included 8 centers, 2 regional and 6 metropolitan, across 3 Australian states between August 2015 and May 2019. Two regional centers withdrew after completing the control phase because of a lack of resources or slow patient recruitment. The remaining results focus on the remaining 6 centers (Figure 1).

A total of 8099 patients were screened from whom 1564 were eligible for either primary or secondary outcome measures. Of these, 754 patients gave consent to participate in the study. These 6 centers were randomized to receive the intervention in order resulting in 359 patients in the control phase and 329 in the intervention phase, with 544 contributing to primary outcome measurement. In terms of the missing values in the follow-up period (ie, week 2 and week 4), the patterns of missing were not associated with the main demographic variables suggesting missing completely at random.

### Baseline Characteristics

Baseline characteristics for samples of patients contributing data to primary and secondary outcomes are summarized in Table 1. For NRS scores of 5 or more, there was a higher proportion of males in the control group than the intervention group (150 of 280 participants [54%] vs 121 of 264 participants [46%]) and a higher proportion of people with breast cancer in the intervention group 61 of 264 participants (23%) vs 46 of 280 participants (16%) in the control group. However, there were no statistically significant differences in the characteristics of patients between phases.

**Table 1.** Baseline Patients’ Characteristics at Control and Intervention Phases and Comparisons of the Total Between Phases (N = 688)^a^

Characteristic	Participants, No. (%)					
	Control phase (n = 359)			Intervention phase (n = 329)		
	NRS≥5 (n = 280)	NRS 2-4 (n = 79)	Total	NRS≥5 (n = 264)	NRS 2-4 (n = 65)	Total
Male	150 (54)	46 (58)	196 (55)	121 (46)	34 (52)	155 (47)
Female	130 (46)	33 (42)	163 (45)	143 (54)	31 (48)	174 (53)
Age, mean (SD)	63.9 (12.6)	65.1 (11.5)	64.2 (12.1)	63.4 (12.9)	64.0 (11.7)	63.6 (12.7)
NESB	23 (8)	0	23 (6)	27 (10)	0	27 (8)
Cancer type						
Breast	46 (16)	16 (20)	62 (17)	61 (23)	10 (15)	71 (22)
Lung	33 (12)	13 (16)	46 (13)	35 (13)	7 (11)	42 (13)
Gastrointestinal	53 (19)	10 (13)	63 (18)	47 (18)	16 (25)	63 (19)
Genitourinary	48 (17)	19 (24)	67 (19)	44 (17)	12 (18)	56 (17)
Head and neck	21 (8)	3 (4)	24 (7)	11 (4)	3 (5)	14 (4)
Hematologic	8 (3)	2 (3)	10 (3)	10 (4)	2 (3)	12 (4)
Others	50 (18)	14 (18)	64 (18)	44 (17)	13 (20)	57 (17)
Not recorded	21 (8)	2 (3)	23 (6)	12 (5)	2 (3)	15 (5)
Worst NRS score, mean (SD)	6.8 (1.5)	3.1 (0.9)	5.0 (2.6)	6.8 (1.4)	3.0 (0.8)	4.9 (2.6)
Mean NRS score, mean (SD)	5.9 (1.2)	2.9 (0.8)	3.5 (2.1)	5.9 (1.0)	2.9 (0.8)	3.2 (2.1)

Abbreviations: NESB, Non–English-speaking background; Not recorded, diagnosis was not recorded; NRS, numeric rating scale.

^a^ No comparison results were statistically significant.

**Figure 2.**
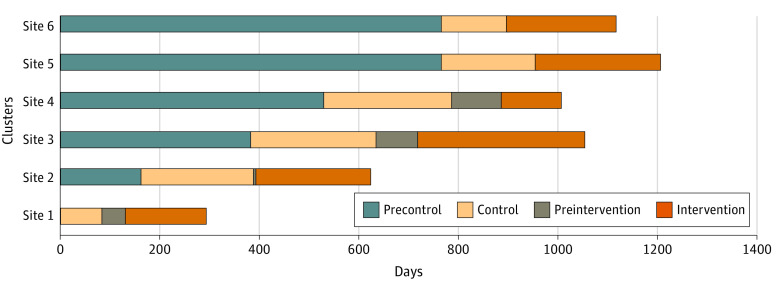
Stepped Wedge Cluster Timelines

The duration of phases varied between centers, with the control phases lasting between 84 and 255 days and the intervention phases between 120 and 338 days. Initially, the training phase was planned to last 3 weeks. However, this was not possible at 2 centers because of time constraints, with the result that they transitioned from control to intervention phases within a few days only. The sustainability phase was also abandoned because of lack of resources at centers to continue the intervention after resourcing from the project team was removed. The stepped-wedge timeline is shown in Figure 2.

### Outcomes

For the primary outcome, namely a reduction in the pain score by 30% among those with a score of 5 or more on the NRS on worst pain at week 1, there was no significant difference between the control and intervention phases. The proportions were similar with 31 of 280 participants (11.9%) in the control phase and 30 of 264 participants (11.8%) in the intervention. After adjusting for the potential confounding variables, the results indicated no significant increase in the odds of the outcome (OR, 1.12; 95% CI, 0.79-1.60). Results on the secondary outcome measures are also summarized in Table 2. As shown, there were no significant differences in the outcome measures between phases. Regarding the secondary outcome measures heiQ and CES, the responses were small with many missing data rendering the analyses unfeasible.

**Table 2.** Results Obtained on the Primary and Secondary Outcomes

Outcomes of the study	Control phase, mean (SD)	Intervention phase	Results on comparison, Z-value^a^	*P* value
Primary outcome				
Pain score reduction of 30% among those with ≥5 NRS on worst pain at week 1	31 (11.9)	30 (11.8)	OR, 1.12 (95% CI, 0.79-1.60)	.51
Secondary outcome				
Worst pain NRS score at week 1 follow-up mean (SD)	4.4 (2.4)	4.3 (2.3)	0.28	.78
NRS score at week 1 follow-up, mean (SD)	3.0 (1.9)	2.5 (1.8)	−0.96	.34
Total QLQ-C15-PAL score at week 1 follow-up	14.6 (1.9)	14.6 (1.8)	0.11	.92
Worst pain NRS score at week 2 follow-up, mean (SD)	4.7 (2.7)	4.2 (2.4)	−0.89	.37
NRS score at week 2 follow-up, mean (SD)	3.1 (2.0)	2.7 (1.8)	−0.70	.48
Total QLQ-C15-PAL score at week 2 follow-up, mean (SD)	14.5 (2.2)	14.9 (1.8)	1.82	.07
Worst pain NRS score at week 4 follow-up, mean (SD)	4.4 (2.5)	4.3 (2.7)	0.36	.72
NRS score at week 4 follow-up, mean (SD)	2.9 (1.7)	2.8 (2.1)	−0.58	.55
Total QLQ-C15-PAL score at week 4 follow-up, mean (SD)	14.8 (1.9)	14.7 (1.8)	−0.11	.91

Abbreviations: NRS, numeric rating score; OR, odds ratio; QLQ-C15-PAL, Quality of Life Questionnaire Core 15 Palliative Care.

^a^ Adjusted for cluster effect, age, sex, cancer type, Qstream, information pack, audit cycles, and time spent in the phase.

Changes in pain scores within each phase are reported in Table 3. Mean average pain improved by an SD of 0.5 or more at all time points in both phases except week 1 in the intervention phase. Mean worst pain improved by 0.5 SD at week 4 in both phase. Table 3 shows the changes in mean (SD) NRS scores from baseline at different time points of follow-up by phases and results on pairwise comparisons.

**Table 3.** Changes in Pain NRS Scores From Baseline at Different Time Points of Follow-up by Phases and Results on Pairwise Comparisons

Week of follow-up	Changes in scores for participants in control phase, mean (SD)		Changes in scores for participants in intervention phase, mean (SD)	
	Worst score	Average score	Worst score	Average score
1	–0.70 (2.44)^a^	–2.15 (2.18)^a^	–0.01 (2.27)	–1.04 (2.32)^a^
2	–0.70 (2.67)^a^	–2.32 (2.38)^a^	–0.43 (2.17)	–1.93 (2.09)^a^
4	–0.89 (2.54)^a^	–2.41 (2.40)^a^	–0.88 (2.67)^a^	–2.36 (2.55)^a^

^a^ *P* < .001.

Fidelity to the intervention was lower than anticipated. Two sites had 2 audit cycles and the other sites had 1. Completion rates of health professional training varied between sites from 12% to 74%. The proportion of patients receiving written information (5 sites) was 30% (20%-44%) vs 22% (2%-30%) in the control arm.

## Discussion

In this adequately powered, stepped wedge, cluster randomized trial, guideline implementation strategies at the level of the clinical service, health professional, and patient did not deliver a 30% or greater improvement in patients’ pain compared with usual care. This finding was despite the combined use of a conceptual framework,^26^ evidence-based implementation strategies, and services with clinical champions.^15,16,27,31^ Participants in both phases reported improved mean and worst pain scores over time. However, the lack of a comparison group where no screening was offered means we cannot conclude whether this improvement was because of screening, usual pain management, or a Hawthorne effect.^32^

There are several potential explanations for the lack of effect difference between groups. Interventions added to usual care are inadequate without dedicated resourcing and focus. The fidelity to the intervention may have been insufficient in terms of the number of rounds of audit and feedback and proportions of staff and patients who completed the spaced education module. The implementation of these strategies as a multicomponent intervention may have required a more intensive approach and more effective engagement with the cancer service management than was achieved. We had used a behavior change framework to design the interventions.^26^ The use of an explicit implementation framework, such as PARIHS (Promoting Action on Research Implementation in Health Services), would likely have helped us to better understand and act upon the implementation issues arising from this evaluation trial as it was conducted.^33^ Centers participating had high baseline knowledge of pain guidelines and high proportions of complex patients with refractory pain. Barriers to pain management previously identified, including participation in pain management intervention being seen as a lower priority and related trials as less important a contribution to the knowledge base than those concerned with curative treatment, may have been contributing factors.^19^ The team is currently analyzing qualitative data collected during the trial to better understand the challenges to fidelity and how this might be improved in the future.

Our finding that pain improved during both phases mirrors the findings of at least one other study that compared screening alone with screening plus a pain treatment protocol.^34^ Optimistically, it may be that improvements in the screening condition resulted from clinicians using the data during consultations without the need for prompting introduced during the Intervention phase. However, previous randomized clinical trials of screening interventions vs no screening in outpatient services have rarely demonstrated improvements in pain scores, especially in palliative care.^35^ Also, the current study used a paper-based screening system, which has largely been replaced in more recent literature by electronic systems due to their better potential for integrating with electronic health records and therefore influencing processes of care.^36^ We used paper-based screening because technical problems with electronic screening encountered when piloting the intervention at a single center suggested it would not be feasible to implement across multiple centers during the study period.^9^ Additionally, patient recall over weeks may vary.^37^ The strategies targeted the service but not the health system policies and procedures for the collection of PROMs, and this system-level change is needed for embedding screening into routine care.

Given evidence that collecting and acting on (PROMs) improve outcomes,^38^ the incidental observation that these were not routinely collected in cancer centers requires further attention. The PROM collection was seen as research rather than a clinical practice tool and changing this mindset among clinicians is an important direction for person-centered care.^39^

Patient education is effective in pain management and should be embedded in workflow for cancer patient care.^40^ A major barrier to optimal patient education was the lack of nurses in ambulatory care. Adequate staffing with nurses in ambulatory care would provide rapid, focused assessment of patients, develop long-term nurse, patient, and family relationships, and deliver patient education.^41^

### Limitations

This study had limitations. Methodological limitations included the fact that there was poor participation for secondary outcomes at week 1 because many potential participants did not receive the information and consent documents by mail in the requisite time frame. The fact that center staff and the research team could not be blinded may have biased the patient selection and outcome data collection. Response bias was less likely because patients were only minimally aware of the study aims and design. There was also potential for selection bias because of patients who chose not to complete screening or found rating pain difficult with numeric rather than categorical scales. It may be that phone collection of PROMs resulted in different results than in-person collection; however, studies in other populations have found comparable results.^42^

## Conclusions

In this study, pain management guideline implementation strategies did not significantly improve pain-related outcomes for outpatients with advanced cancer compared with usual care. This likely reflects limited intervention uptake by centers, perhaps because of a lack of additional dedicated resources. Oncology ambulatory care services require adequate resources to provide routine pain screening, patient and staff education, and quality improvement cycles.

## Notes

Supplement 1.
Trial Protocol

Supplement 2.
Data Sharing Statement
