# Supplementary material for: Effect of Cancer Pain Guideline Implementation on Pain Outcomes Among Adult Outpatients With Cancer-Related Pain: A Stepped Wedge Cluster Randomized Trial
Source: JAMA Netw Open. 2022 Feb 21;5(2):e220060. doi: 10.1001/jamanetworkopen.2022.0060 (PMC8861847; doi:10.1001/jamanetworkopen.2022.0060)
Supplement: Supplement 1. — Trial Protocol [file jamanetwopen-e220060-s001.pdf]

The Stop Cancer PAIN Trial:
A guideline implementation study

TITLE:	Implementing clinical practice guidelines for cancer pain in adults to ensure equitable, cost-effective, evidence-based, person-centred care: A phase III pragmatic stepped wedge cluster randomised controlled trial of guidelines and screening with implementation strategies versus guidelines and screening alone to improve pain in adults with cancer attending outpatients oncology and palliative care centres
PROTOCOL NUMBER	I001/ V5.1
LEAD STUDY INVESTIGATOR	Prof Melanie Lovell<br>Consultant Palliative Care Physician, HammondCare; Senior Lecturer, Sydney Medical School, The University of Sydney<br>E: mlovell@hammond.com.au P: +612 8788 3953 F: +612 9903 8383<br>97-115 River Road, Greenwich NSW 2065 Australia
DATE OF PROTOCOL	4th September, 2018
STUDY SPONSOR	The University of Sydney

Confidentiality Statement

Information in this protocol should not be disclosed, other than to those involved in the execution or ethical review of the study, without written authorisation from the lead investigator.

Investigator Team

Chief Investigators			
**Name**	**Organisation**	**Telephone**	**Qualifications**
A/Prof Melanie Lovell	HammondCare, Greenwich Hospital, Greenwich	02 8788 3953	MBBS, PhD, FRACP, FAChPM
Prof Jane Phillips	Centre for Cardiovascular and Chronic Care, University of Technology Sydney, Ultimo	02 9514 4822	RN, BN, PhD, MRCNA
Prof Meera Agar	HammondCare, Braeside Hospital, Wetherill Park	02 9616 8654	FRACP, FAChPM, MPC, MBBS, PhD
Prof Frances Boyle	The Patricia Ritchie Centre for Cancer Care and Research, Mater Hospital, North Sydney	02 9900 7300	MBBS, FRACP, PhD
Prof Patricia Davidson	Centre for Cardiovascular and Chronic Care, University of Technology Sydney, Ultimo<br><br>Department of Acute and Chronic Care, Johns Hopkins University, Baltimore, USA	02 9514 4822	PhD, MEd, RN, FAAN,
Dr Tim Luckett	Improving Palliative Care through Clinical Trials (ImPaCCT), University of Technology Sydney, Ultimo	02 9514 4861	BSc, PhD
Prof David Currow	Palliative and Support Services, Flinders University, Adelaide	08 7221 8235	BMed, MPH, FRACP
Prof Lawrence Lam	Faculty of Health and Graduate School of Health, University of Technology Sydney, Ultimo	02 9514 4242	B.Sc. (Hons), MAppPsy, MPH, Grad Dip Biostats, PhD, FACE
Dr Nikki McCaffrey	Deakin Health Economics, School of Health & Social Development, Deakin University, Burwood, Victoria	03 9246 8767	BSc (Hons), MSc, PGDip(HEc), PGDip(ClinPharm)
Prof Tim Shaw	Workforce Education and Development Group (WEDG), University of Sydney, Camperdown	02 9351 5181	BSc (Hons), PhD

Site investigators			
**Name**	**Site**	**Telephone**	**Qualifications**
Prof Meera Agar	Braeside Hospital, Wetherill Park, NSW	02 9616 8654	FRACP, FAChPM, MPC, MBBS, PhD
Dr Peter Allcroft	Southern Adelaide General Repatriation Hospital, Adelaide	(08) 8275 1732	MBBS, MPM, FRACP
Dr Gregory Barclay	Illawarra Shoalhaven Cancer and Haematology Network (ISCaHN) – Illawarra Shoalhaven Local Health District	02 4223 8380	MBBS FRACGP, FAChPM
Dr Ankit Jain	Mid North Coast Cancer Institute, Coffs Harbour, NSW	(02) 6581 4053	MBBS, MD, DM
Dr Katherine Allsopp	Crown Princess Mary Cancer Centre, Westmead, NSW	02 9845 5200	MBBS BSc(Med) FRACP FAChPM
Dr Caitlin Sheehan	St George Hospital and Calvary Health Care Sydney, Sydney, NSW	(02) 9553 3111	BMed, FRACP, FAChPM
Prof Martin Stockler	Concord Repatriation General Hospital, Concord, NSW	02 9562 5313	MBBS (Hons), MSc, FRACP
Prof Rick Kefford	Macquarie Private Hospital, Macquarie Park, NSW	02 9850 2766	MB BS PhD FRACP
Prof Stephen Clarke	Royal North Shore Hospital, St Leonards, NSW	02 9926 5048	MBBS FRACP
Dr Mamta Bagia	Northern Beaches Cancer Service, Manly, NSW	02 9976 9611	MBBS FRACP
Dr Alex Clinch	Peter MacCallum Cancer Centre, East Melbourne, VIC	03 8559 7960	MBBS FRACGP FAChPM
Prof Bogda Koczwara	Flinders Medical Centre, Bedford Park, SA	08 8204 8997	BMBS, FRACP, MBioethics, FAICD
Dr Sarah Wenham	Far West Local Health District Cancer and Palliative Care, NSW	08 8080 1333 (switch)	MBChB MRCP(UK) FRACP FAChPM
Dr Jessica Lee	Concord Repatriation General Hospital, Concord, NSW	02 9767 6799	MBBS FRACP FAChPM

Protocol History			
**Version**	**Date**	**Author**	**Reason**
1.0	14/10/2014	Melanie Lovell	New protocol from draft
1.1	09/03/2015	Melanie Lovell	Addition of investigators and sites
2.0	01/05/2015	Melanie Lovell	Change to sites, sample size estimate, addition of 5 quality of life survey items to enable cost-utility analysis, and confirmation that sites can continue to use an existing screening tool where this is working well
2.1	27/07/2015	Melanie Lovell	Change to enable project staff to assist local site staff in collating and entering screening data and demographic and contact details; inclusion of cancer type as a self-reported item on the screening survey; removal of cancer stage from the screening dataset; clarification of written informed consent process
3.0	11/11/2015	Melanie Lovell	Change of wording to baseline screening tool and removal of quality of life measures at baseline;<br>Change to cost effectiveness analysis;<br>Clarification of patient eligibility in relation to diagnosis of cancer;<br>Clarification of the role of interpreters;<br>Addition of a letter of approach to patients;<br>Small changes to wording within the letter of approach to carers;<br>Amendments to PISCFs to reflect above changes and to add script about Medicare and Pharmaceutical Benefits Scheme (PBS) information about data collection, as per the requirements of the Australian Government Department of Human Services;<br>Participant information sheets, consent forms and letters of approach have been removed as Appendices to allow for version changes that have occurred and may occur throughout the duration of the trial.
3.1	17/12/2015	Melanie Lovell	Change to the processes of data entry by site staff and secure transfer of baseline screening data from study sites to the project team.
4.0	16/08/2016	Melanie Lovell	Change to consent processes for patient- and caregiver secondary data;

			Addition of a sustainability phase (including change to Figure 1);<br>Updates to investigator titles;<br>Removal of reference to consent withdrawal form attached pg. 28 of protocol_V3.1 to adhere to HREC documentation
5.0	05/04/2017	Melanie Lovell	Request for word change to pain screening document to identify where the project team came from and variation in information required by health professional;<br>Change wording in protocol section 9.2 Appendix (pg 44) to reflect new wording on screening form;<br>Add new site investigators and update place of work for investigators;<br>Remove erroneous words from the document on page 45
5.1	22/08/2018	Melanie Lovell	Update the names of the site investigators;<br>Update of protocol history

TRIAL SUMMARY

Background

Pain is a common and distressing symptom in people with cancer that is under-treated. Better management of pain has the potential to improve quality of life for most people with cancer and reduce healthcare costs by avoiding unnecessary hospital admissions, supporting non-pharmacological approaches and quality use of medicines, and reducing caregiver distress.

International studies have found that guidelines and clinical pathways can improve the quality of care for cancer pain and patient outcomes, but implementation is challenged by barriers at the levels of the healthcare system, provider and patient. Evidence supports implementation of guidelines by means of health professional and patient education and audit and feedback.

Aim

To evaluate the effectiveness and cost-effectiveness of a suite of strategies designed to implement Australian clinical practice guidelines to improve pain outcomes in adult outpatients with cancer in metropolitan, regional and rural oncology and palliative care settings.

Study design

The trial will use a stepped-wedge cluster randomised controlled design wherein participating services will take turns in introducing the strategies so that outcomes can be compared before and after the change in practice.

A qualitative sub-study will use semi-structured interviews and focus groups to investigate aspects of the intervention that have worked well or could be improved from the perspectives of patients, carers and centre staff.

Control and intervention arms

Control arm

Whilst in the control arm, participating centres will be provided with an electronic or paper-based system for screening patients for worst and average pain severity via a 0-10 numerical rating scale (NRS). Centres that are already using a different tool for screening symptoms will continue to use the same tool where this is preferred, with addition of screening items for pain as necessary. Screening tools and patient-held resources will be translated to support communication by non-English speaking patients, who are at special risk for poor outcomes. Staff at the centre will be at liberty to use the pain screening data in any way they wish.

Intervention arm

During a training phase, designated 'champions' and staff at each centre will attend workshops to learn about the project and how to tailor implementation strategies to their centre's needs. Implementation strategies will include:

1. An audit tool to enable centres to monitor how well they are implementing core standards of cancer pain assessment and management;
2. Tools for identifying barriers and facilitators to cancer pain screening, assessment and management and strategies to overcome barriers;
3. QStream online health professional education to test and consolidate knowledge of cancer pain assessment and management;
4. Patient-held resources to ensure management remains centred on individual patient needs, support patient-health professional communication and coordination and help patients advocate for evidence-based, person-centred care;

In the intervention phase, implementation strategies will be put in place and monitored. A community of practice between staff at different centres implementing the strategies will be facilitated by means of regular teleconferences, chaired by a project team member.

Sites and participants

Sites will be outpatient services at oncology and palliative care centres. Participants will include patients, carers and centre staff at each site.

Inclusion criteria for patients to be included in the primary endpoint will be: 1) attending a participating cancer centre as an outpatient during the study period; 2) having a diagnosis of current active cancer; 3) being able to self-complete a 0-10 NRS for severity of worst and average pain in English, Chinese, Italian, Greek, Vietnamese or Arabic; 4) choosing not to opt-out of being contacted 1 week later to complete the NRS over the telephone and giving verbal consent to do so when telephoned; 5) a score of moderate-severe (≥5) on the NRS for worst pain.

Inclusion criteria for patients contributing to secondary outcomes will be meeting inclusion criteria 1, 2, 3 and 4 above and also: 5) a score ≥2 on the NRS for worst pain; 6) providing verbal informed consent in English; 7) having spoken and written English proficiency sufficient to complete study measures.

Inclusion criteria for carers will be: 1) being identified by a patient who has given verbal informed consent to participate in the study as providing them with substantial emotional and practical support in an unpaid capacity; 2) providing verbal informed consent; 3) having spoken and written English proficiency sufficient to complete a brief survey and/or interview.

Inclusion criteria for centre staff will be: 1) being employed on a permanent basis either full- or part-time at a participating oncology or palliative care centre in a role that provides clinical care to patients with cancer pain or front desk, client-focused administrative support; 2) providing written informed consent.

Objectives

Primary objective

To evaluate the ability of the intervention versus control arm to increase the probability that patients screened as having moderate-severe (≥5) worst pain will experience a clinically important improvement of 30% on a 0-10 NRS 1 week later.

Secondary objectives

1. To evaluate the ability of the intervention arm versus the control arm to:

    i. improve mean worst and average pain severity in patients screened as having clinically significant (≥2) worst pain from time of screening to 1, 2 and 4 week later by half a standard deviation (0.5 SD).

    ii. improve patient empowerment in patients screened as having clinically significant (≥2) worst pain at 1, 2 and 4 weeks post-screening by 0.5 SD on the Health Education Impact Questionnaire (heiQ) (2).

    iii. improve the experiences of unpaid carers of participating patients at 2 and 4 weeks by 0.5 SD on the Carer Experience Scale (CES) (3).

2. To compare the mean patient quality of life (QOL) at 1, 2 and 4 weeks post-screening between the intervention and control arm.

3. To test the cost-effectiveness of the implementation package versus control arm.

4. To inform understanding of the mechanisms by which outcomes are improved to inform ongoing refinement and tailoring of the complex intervention to different service settings and patient populations in the future.

Sample size

100 patients with score 5+ on a numerical rating scale for pain at screening recruited at Week 1 for worst pain score (50 during the control arm and 50 during the intervention arm) per site, across eight sites.

Analysis

For the main analysis, linear mixed models (5) will be used to model the outcomes of interest, while accounting for the clustering and longitudinal design. Sensitivity analyses will be used to explore the effects of the intervention for different sub-groups and to explore objective 3.
The primary outcome for cost-effectiveness analysis will be the incremental cost per additional responder at the end of Week 1 (response is defined as a clinically important improvement of 30% on a 0-10 NRS 1 week post-screening for those with moderate-severe (≥5) worst pain). Quality of life data collected at weeks 1, 2 and 4 will be used to conduct a modelled cost-utility analysis. As economic data may be skewed, confidence intervals will be estimated with bootstrap methods (6). Sensitivity analysis will examine the effect of assumptions and determine which cost components drive the results.

Thematic coding and classifying techniques will underpin the analysis of semi-structured interviews and focus groups (7).

CONTENTS

TRIAL SUMMARY **5**
CONTENTS **9**
LIST OF ABBREVIATIONS **11**
BACKGROUND AND RATIONALE **12**
1.1 REVIEW OF THE LITERATURE 12
1.2 WORK BY THE CURRENT TEAM 13
1.3 RATIONALE FOR INTERVENTION 15
1.4 SIGNIFICANCE 16
2.0 STUDY OBJECTIVES **17**
2.1 AIM 17
2.2 OBJECTIVES 17
2.2.1 PRIMARY OBJECTIVE 17
2.2.2. Secondary objectives *17*
3.0 STUDY POPULATION **18**
3.1 TARGET POPULATION 18
3.2 INCLUSION CRITERIA 18
3.2.1 PATIENTS **18**
3.2.2 UNPAID CARERS 18
3.2.3 CENTRE STAFF **19**
3.3 EXCLUSION CRITERIA 19
3.3.1 Patients *19*
3.3.2 Unpaid carers *19*
3.3.3 Centre staff *19*
INVESTIGATIONAL PLAN **20**
3.4 STUDY DESIGN 20
3.4.1 Figure 1. Stepped wedge design with staggered introduction of training/intervention in 8 centres *20*
3.5 RATIONALE FOR STUDY DESIGN 20
3.6 RANDOMISATION 21
3.7 BLINDING 21
4.0 CONTROL AND INTERVENTION ARMS **22**
4.1 DESCRIPTION 22
4.1.1 Control arm *22*
4.1.2 Intervention arm *22*
4.2 CONCURRENT TREATMENTS 23
4.3 POST STUDY TREATMENTS 23
5.0 STUDY PROCEDURES **23**
5.1 IDENTIFYING AND RECRUITING PARTICIPANTS DURING INTERVENTION PERIOD 23
5.1.1Patients *23*
..... 24
..... 24
5.1.2 Figure 2: Study diagram of patient recruitment and data collection for primary endpoint of worst pain at 1 week after positive screen *25*
..... 25
5.1.2 Figure 3: Study diagram of patient and carer recruitment and data collection for secondary outcomes *25*
5.1.3 Unpaid Carers *28*
5.1.4 Centre staff *28*
5.1.5 Withdrawal *28*
5.2 OUTCOMES AND MEASURES 29
5.2.1 Primary outcome *29*

5.2.2 Table 1: Patient and carer measures ... *29*
5.2.3 Secondary outcomes ... *30*
5.2.4 Structural and process measures ... *31*
5.2.5 Descriptive/control variables ... *32*
5.3 QUALITATIVE SUB-STUDY ... 33
5.4 SAFETY MONITORING ... 33
5.4.1 Reporting of incidents ... *33*
5.4.1.1 Follow-up of incidents ... *33*
5.4.2 Stopping rules ... *33*
5.4.3 Monitoring ... *33*
5.5 STATISTICS ... 34
5.5.1 Statistical analysis ... *34*
5.5.2 Sample size and power calculations ... *34*
5.5.3 Cost-effectiveness analysis ... *34*
5.5.4 Qualitative analysis ... *35*
6.0 ETHICS ... **35**
6.1 BENEFIT ANTICIPATED FROM THE STUDY ... 35
6.2 THE POSSIBILITY OF PSYCHOLOGICAL STRESS ... 35
6.3 RESEARCH ON PEOPLE IN DEPENDENT RELATIONSHIPS ... 36
6.4 METHOD AND NATURE OF RECRUITMENT AND ADVERTISING ... 36
6.5 PROTECTION OF PRIVACY AND PRESERVATION OF CONFIDENTIALITY ... 36
6.6 RESTRICTION OF USE OF DATA ... 36
6.7 USE OF PERSONAL INFORMATION ... 36
6.8 ESTIMATED TIME OF RETENTION OF PERSONAL INFORMATION AND PLANNED DISPOSAL ... 37
7.0 STUDY ADMINISTRATION ... **37**
7.1 DATA HANDLING AND RECORD KEEPING ... 37
7.1.1 Direct access to source data ... *37*
7.1.2 Data collection ... *37*
7.1.3 Electronic recording ... *38*
7.1.4 Data entry ... *38*
7.1.5 Data storage ... *38*
7.2 QUALITY CONTROL ... 38
7.2.1 Training procedures: ... *38*
8.0 REFERENCES ... **40**
9.0 APPENDICES ... **44**
9.1 APPENDIX 1: BEHAVIOUR CHANGE FUNCTIONS IDENTIFIED BY A SYSTEMATIC REVIEW BY MICHIE ET AL (30) AND ASSOCIATED STRATEGIES FOR OVERCOMING BARRIERS TO CANCER PAIN ASSESSMENT AND MANAGEMENT AT THE LEVELS OF PATIENT, HEALTH PROFESSIONAL AND HEALTH SYSTEM (ADAPTED FROM (25)) ... 44
9.2 APPENDIX 2: WORDING AT END OF SCREENING GIVING PATIENTS OPPORTUNITY TO OPT OUT OF BEING CONTACTED FOR RESEARCH PURPOSES ... 45
9.3 APPENDIX 3: SCRIPTS FOR APPROACHING PATIENTS AT 1 WEEK ... 45
9.3.1 Primary endpoint data ... *45*
9.3.2 Secondary endpoint data ... *45*
9.4 APPENDIX 4: EMAIL CIRCULAR TO CENTRE STAFF WITH INVITATION TO PARTICIPATE IN ANONYMOUS ONLINE SURVEY ... 47
9.5 APPENDIX 5: ANONYMOUS ONLINE SURVEY QUESTIONS TO STAFF ... 47
9.6 APPENDIX 6: EMAIL CIRCULAR INVITING PARTICIPATION IN STAFF FOCUS GROUPS ... 47
9.7 APPENDIX 7: SURVEY TOOLS ... 48
HEALTH EDUCATION IMPACT QUESTIONNAIRE ... 51
9.8 APPENDIX 8: INTERVIEW GUIDE FOR PATIENTS AND CAREGIVERS ... 54
9.9 APPENDIX 9: FOCUS GROUP/INTERVIEW GUIDE FOR CENTRE STAFF ... 54
9.10 APPENDIX 10: INCIDENT REPORTING TEMPLATE ... 55

LIST OF ABBREVIATIONS

AKPS	Australian Karnofsky Performance Scale
CES	Carer Experience Survey
CRF	Case report form
DOB	Date of birth
ECOG	Eastern Cooperative Oncology Group Performance Status
EORTC QLQ-C15-PAL	European Organisation for the Research and Treatment of Cancer Quality of Life Questionnaire for Palliative Care
EORTC QLQ-C30	European Organisation for the Research and Treatment of Cancer Quality of Life Questionnaire for Cancer
EORTC QLQ-U	European Organisation for the Research and Treatment of Cancer Quality of Life Questionnaire Utility measure
heiQ	Health Education Impact Questionnaire
HREC	Hospital Research Ethics Committee
ImPaCCT	Improving Palliative Care through Clinical Trials
MBS	Medicare Benefits Scheme
PBS	Pharmaceutical Benefits Scheme
PISCF	Participant information sheet and consent form
NRS	Numerical rating scale
QOL	Quality of life

BACKGROUND AND RATIONALE

1.1 Review of the literature

In 2007, 339,000 Australians – or 1.6% of the population - had been diagnosed with cancer within the last 5 years (8). As cancer treatments evolve, those who previously would have died within months are now living with cancer as a chronic condition often in addition to other co-morbidities, with a heavy burden of physical and psychological symptoms (9). Most people with advanced cancer, in particular, present with symptoms related to both cancer and its treatment (10). Symptoms also impact on informal carers, with an estimated 2.6 million Australians providing home care to someone affected by illness or disability, many of whom have cancer.

Pain is experienced by 30-75% of people with cancer and is rated as moderate-severe by 40-50% and severe by 25-30% (11). Studies show that up to 42% have inadequate analgesia (12). Undertreated pain not only reduces patient quality of life (QOL) but also increases health service use, impacts on carers and, at a societal level, reduces the ability of patients and carers to participate actively in employment and other activities. A wealth of literature highlights barriers to pain assessment and management at the levels of patients (e.g. reluctance to report pain, misconceptions regarding opioids), health professionals (e.g. lack of time and expertise) and the healthcare system (e.g. lack of coordination and timely access to analgesia) (13-18). Patients from non-English speaking backgrounds are likely to suffer especially poor levels of care because they are less able to communicate their needs and navigate the healthcare system (19). Patients in regional and rural areas may also not receive the same standard of care as those in metropolitan areas (20), despite the fact that minimum standards for pain assessment and management can be readily achieved without input from specialist services in the overwhelming majority of cases where pain is not complex. These standards include routinely screening and assessing pain, providing patients with pain self-management strategies, ensuring availability of regular and breakthrough analgesia, and offering laxatives to patients receiving opioids (21). Studies have shown that implementation of evidence-based clinical practice guidelines for pain recommending these standards can improve the processes of care and patient outcomes (22).

In Australia, pain in people with cancer has been identified as an important area for improvement by both the National Institute of Clinical Studies (NICS) and the Cancer Institute New South Wales. The recently launched Australian National Pain Strategy has six major goals including, 'timely access to best-practice, evidence-based assessment and care' (23). The Pain Strategy was developed in 2010 at a National Pain Summit and included input from a *Cancer Pain and Palliative Care Working Group* chaired by the current lead investigator. This Group concluded that a primary objective should be promotion of guidelines and systems to ensure adequate assessment and management of pain in people with cancer.

As well as evidence-based, pain management needs to be person-centred. The experience of pain and meaning attributed to it vary substantially between different individuals (13). People often present with comorbid health conditions and other symptoms. Their priorities and preferences also vary according to other commitments in everyday life. For example, people with cancer pain commonly seek a balance between pain and the side-effects of medication, altering dose according to their activities on a day to day basis (24). Patients can be appropriately considered experts in their own pain, both in its experience, exacerbating and ameliorating factors, and in their pain-related needs within the context of their wider lives (25). This means that the role of supportive care might be best thought of as supporting self-management by patients and carers, providing the necessary information, resources and expert advice. Unfortunately, however, patients often report that they get

‘lost in the cracks’ of the health system, and that their care is centred more around the different services they encounter than their needs.

In summary, a comprehensive, systems-based approach is needed to ensure evidence-based assessment and management of pain is implemented across settings and geographical locations for people with advanced breast and other cancers. At the same time, however, special attention is needed to ensure that systems approaches do not lose sight of individual patients.

A systematic review identified three models that have been shown to succeed at least to some degree in implementing evidence-based care for cancer pain (22): 1) institutional models, which provide policies and procedures for regular pain assessment and standardisation of pain treatment; 2) clinical pathways, which provide step-by-step guidance on optimal sequencing and timing of assessment and management; and 3) expert consultation. The reviewers concluded that a clinical pathway combined with a consultation model is currently the optimal method for supporting the most important features of pain assessment and management, namely patient education, an interdisciplinary approach and continuity of care.

A number of systematic reviews have found computerised symptom screening cancer settings to be feasible and acceptable and to contribute to improved processes of care such as doctor-patient communication and referrals (26-28). Evidence is less consistent and compelling for impacts on symptom severity or QOL, suggesting that structured interventions of the kind to be used in the current study are required to ensure screening results are used to optimally inform management.

1.2 Work by the current team

The current project represents the culmination of a program of work by evaluating the efficacy and cost-effectiveness of a comprehensive package to implement best practice pain screening, assessment and management by means of Australian guidelines and a summary of these in clinical pathway form together with a suite of implementation resources developed by the team.

Following the 2010 Australian National Pain Summit, CIA A/Prof Lovell and other members of the Cancer Pain and Palliative Care Working Group and current team adapted international guidelines for cancer pain management for the Australian setting. The resulting guideline has been made available on the Cancer Council Australia Cancer Guideline Wiki (http://wiki.cancer.org.au/australia/Guidelines:Cancer_pain_management), has undergone a period of public consultation, and has been endorsed by the National Pain Summit, the Clinical Oncological Society of Australia (COSA) and Painaustralia.

As part of the guideline development, the team has completed a systematic review and synthesis of qualitative studies on barriers and facilitators to cancer pain management (13). This review highlighted the need to: integrate patient and family education within improved communication; individualise care; consider more non-pharmacological strategies; empower patients and families to self-manage pain; and reorganise multidisciplinary roles around patient-centred care.

A further systematic review and meta-analysis by this team examined the specific components of patient education and training associated with improved pain management, with a special focus on patient-held materials and resources (29). This review took a theory-based approach to meta-analysis using behaviour change ‘functions’ identified by a systematic review by Michie and colleagues (30). It found that educational interventions which included strategies for boosting patients’ sense of control (e.g. question prompt lists) and tailoring management to individual needs were most effective.

The results of the two reviews together were used to inform development of patient-held resources to help patients self-manage their pain, support coordination and communication between health professionals by means of a patient-held record, and advocate for evidence-based person-centred care (e.g. via a symptom diary). These patient-held resources have been designed to complement and support the guidelines implementation, working in tandem to overcome barriers to better pain management at both patient and clinician levels.

The patient-held resources has been designed for use in conjunction with an education booklet and DVD designed by CIs Lovell and Boyle called "Overcoming cancer pain", which is available from the Cancer Council New South Wales (http://www.cancercouncil.com.au/wp-content/uploads/2013/05/CAN487_UCPain_ReprintDec10_LoRes.pdf). This booklet includes information on pain, general advice on non-pharmacological and pharmacological management and advice on further support. The booklet and DVD were found to reduce pain versus usual care in a randomised controlled trial (RCT) (31).

The team has also completed an online survey of current practice for cancer pain assessment and management, which attracted 527 respondents from a diverse range of medical, nursing and allied health disciplines across Australia (32-34). Ninety percent of respondents agreed a need for Australian guidelines and implementation strategy across different service settings.

The team has also developed methods for assessing barriers and facilitators to symptom assessment and management with funding from a university grant (manuscript in progress). Findings from this work underscored results from the survey and other research that coordination of care is among the most significant barriers to symptom control. The methods developed by this project are being used to inform an implementation toolkit for services to use in assessing barriers and ways to overcome them in each local setting.

Whilst pain assessment is considered a core specialist palliative care skill, results from a study undertaken by CI Phillips found that even with this setting, there was little documented evidence of systematic screening and assessment practices (35). A national study found that up to 40% of respondents who had moderate-severe pain upon presentation to an Australian specialist palliative care service still had moderate-severe pain at the end of their episode of care (36). These findings highlight the need to implement the pain guidelines in palliative care as well as oncology.

CIs Phillips and Shaw have developed a 'QStream' clinician training module for cancer pain assessment, which has been successfully trialled in the palliative care setting and was associated with increased levels of nurse knowledge and pain documentation and reduced pain intensity (37). QStream (formerly known as Spaced Education) is an on-line learning format that has been shown to significantly improve knowledge and retention of guideline content in RCTs (38). Clinical content questions are delivered to participant's regular e-mail which also provides immediate feedback regardless of whether questions are answer correctly or incorrectly. Incorrect answers are resubmitted to participants at a later date and only retired when they have been answered correctly on two occasions. QStream has been well received in the areas of cancer screening, quality and safety and has been identified as acceptable to general practice. Further modules are being developed by the team to test and improve clinician understanding of cancer pain management. Qstream has been chosen because it is a sustainable and feasible form of education within busy clinical environments, and is applicable to all members of the multi-disciplinary team.

A pilot has been completed at one public palliative care (Greenwich Hospital, Sydney) and one private oncology centre (the Mater Hospital, North Sydney). Initial data have been collected on the

user-friendliness and usefulness of the guidelines via interviews with health professionals and usefulness of the patient-held resources via interviews with patients and carers (39). We have also piloted screening for pain via touchscreen computer and feedback to clinicians, which will be used in the proposed project. Electronic screening for pain and other symptoms is already used in many cancer centres. The pilot has overcome significant barriers to implement screening and feedback of results to treating physicians. A manual has been developed to facilitate implementation at sites participating in the proposed research. The pilot also implemented a clinical audit tool developed by Victoria Health which will be used in the proposed study (40). Data from this audit tool provide useful baseline data to inform the proposed research.

Throughout the above program, the team has actively engaged with clinical and research communities via local, national and international presentations and workshops attended by multi-disciplinary health professionals and consumers.

1.3 Rationale for intervention

The research summarised above has identified the following as important strategies for supporting implementation of evidence-based, person-centred care of cancer pain that will be evaluated in the current study, in addition to the guidelines and screening introduced above.

1. An audit tool to enable centres to monitor how well they are implementing core standards of cancer pain assessment and management;
2. A toolkit for identifying barriers and facilitators to practice change and implementing strategies to overcome these;
3. QStream online health professional education to test and consolidate knowledge of cancer pain assessment and management;
4. Patient-held resources to ensure management remains centred on individual patient needs, support patient-health professional communication and coordination and help patients advocate for evidence-based, person-centred care.

See Appendix 1 for mapping of these strategies against Michie et al’s behaviour change wheel (30).

Fidelity of the above strategies will be underpinned by an implementation manual, staff training, and an ongoing community of practice. The manual will provide guidance on using the above resources to optimum effect in the local setting. Staff training will include a brief (half-day) workshop for all clinical and front-of-house administrative staff aimed at familiarising them with the guidelines and project, as well as more intensive training designed to enable ‘clinical champions’ at each site to administer the strategies above and monitor progress. A community of practice between staff at different centres implementing the strategies will be facilitated by means of regular teleconferences, chaired by a project team member.

This package is a *complex intervention*, as defined by the UK Medical Research Council (MRC) (see the following open source online article for details (41)). Complex interventions involve several components that interact with each other and contextual factors to mediate effect. Complex interventions require tailored implementation to local contexts and monitoring of processes to assess fidelity of implementation, clarify causal mechanisms, and identify influential contextual factors to inform ongoing development and evaluation. The program of work described above represents a *development phase* that has been completed over a number of projects together with a *feasibility and piloting ph*ase that ended in October 2014. The current project is the *evaluation phase* aimed at determining efficacy and cost-effectiveness and understanding change process

within various service settings. This will inform dissemination, surveillance and monitoring, and longer-term follow-up in an *implementation phase* which will follow.

To summarise, research suggests that targeted strategies are needed to support implementation of evidence-based guidelines to ensure best-practice screening, assessment and management of cancer pain. The aim of this project is to test the efficacy and cost-effectiveness of a complex intervention that comprises a suite of resources each of which show promise to support implementation of guidelines to ensure evidence-based, person-centred care for cancer pain.

1.4 Significance

As people with cancer are living longer, addressing symptoms is ever more critical in optimising functioning and QOL, as unrelieved symptoms interfere in all aspects of life – mobility, working, relationships, sleep and life enjoyment. Care practices and standards vary widely across the country, leading to a ‘lottery’ in quality of care and outcomes. More than half of cancer patients report unmet needs for symptom control, information and support. Fear of pain becoming uncontrollable in the terminal phase is prevalent and may contribute to clinical levels of psychological distress.

The proposed project is strategic at a healthcare systems level because it will: 1) facilitate coordination between different sectors involved in caring for people in the community with cancer. Problems with coordination have been consistently emphasised by research looking at barriers and facilitators to cancer care; 2) ease the transition between supportive cancer care and palliative care for people with incurable cancer; 3) take a coordinated approach to overcoming barriers to implementation at systems, provider and patient levels; and 4) ensure that care is firmly centred on the patient and his/her family whilst simultaneously ensuring standardised best practice evidence-based care.

The patient-held resources to be evaluated in this project will empower patients and carers to self-manage pain in the community with the support of health professionals as needed. This model is the mainstay of management for symptoms from other chronic diseases like arthritis and diabetes; now is a strategic time to make the same transition for cancer. Importantly, the project will provide a template that can be used to implement guidelines for other symptoms, including those in which the current team has expertise. The proposed intervention has inbuilt strategies for centres to monitor performance and act on gaps in care, and includes the required educational and systems supports to facilitate change.

If the implementation strategies are found cost-effective, they will be made available free-of-charge on relevant websites (e.g. CareSearch) and distributed to peak bodies (e.g. Cancer Council, COSA and Palliative Care Australia) to facilitate nationwide translation. The clinical practice guidelines will remain on the Wiki platform for people to refer to and submit emerging evidence for updating. A dissemination strategy will be devised by the team in partnership with peak bodies. Consumer advocacy will play an especially important role in promoting use of the guidelines and associated implementation strategies. The team will also make use of its established networks at local health district, state and national levels in both research and practice.

2.0 STUDY OBJECTIVES

2.1 Aim

To evaluate the effectiveness and cost-effectiveness of a suite of strategies designed to implement Australian clinical practice guidelines to improve pain outcomes in adult outpatients with cancer in metropolitan, regional and rural oncology and palliative care settings.

2.2 Objectives

2.2.1 Primary objective

To evaluate the ability of the intervention versus control arm to increase the probability that patients screened as having moderate-severe (≥5) worst pain will experience a clinically important improvement of 30% on a 0-10 numerical rating scale (NRS) 1 week later.

2.2.2. Secondary objectives

1. To evaluate the ability of the intervention arm versus the control arm to:

    i. improve mean worst and average pain severity in patients screened as having clinically significant (≥2) worst pain from time of screening to 1, 2 and 4 week later by half a standard deviation (0.5 SD).
    ii. improve patient empowerment in patients screened as having clinically significant (≥2) worst pain at 1, 2 and 4 weeks post-screening by 0.5 SD on the Health Education Impact Questionnaire (heiQ) (41).
    iii. improve the experiences of unpaid carers of participating patients at 2 and 4 weeks by 0.5 SD on the Carer Experience Scale (CES) (3).

2. To compare the mean patient quality of life (QOL) at 1, 2 and 4 weeks post-screening between the intervention and control arm.

3. To test the cost-effectiveness of the implementation package versus control arm.

4. To inform understanding of the mechanisms by which outcomes are improved to inform ongoing refinement and tailoring of the complex intervention to different service settings and patient populations in the future.

3.0 STUDY POPULATION

3.1 Target population

Adult outpatients with current active cancer, defined as people with residual, locally advanced or solid tumour, who are experiencing pain, and their unpaid carers.

3.2 Inclusion criteria

There are three groups of participants – patients, unpaid carers and centre staff, with the inclusion criteria listed below. There are two patient participant populations for this study, one who will contribute to the primary outcome data and the second who will contribute to secondary outcomes. The methodological details of the two components of the study are outlined under *Consent* below. The study is funded by the National Breast Cancer Foundation of Australia who require the sample to include at least 60% people with breast cancer. Over-sampling of patients with this diagnosis will occur as necessary to meet this target, although it is anticipated that the high prevalence of breast cancer and proven willingness of people with that diagnosis to take part in research may make over-sampling unnecessary.

3.2.1 Patients

3.2.1.1. Inclusion criteria for patients contributing to primary outcome

1) Attend a participating oncology and palliative care centre as an outpatient during the study period
2) Have a diagnosis of current active cancer
3) Be able to self-complete a 0-10 numerical rating scale (NRS) for severity of worst and average pain in English, Chinese, Italian, Greek, Vietnamese or Arabic)
4) Choose not to opt-out of being contacted 1 week later to complete the NRS and give verbal consent to do so.
5) Score ≥5 on the NRS for worst pain

3.2.1.2 Inclusion criteria for patients contributing to secondary outcomes:

Meet inclusion criteria 1, 2, 3 and 4 above and also:

1) Score ≥2 on the NRS for worst pain;
2) Provide verbal informed consent in English;
3) Have spoken and written English proficiency sufficient to complete study measures.

Patients will be eligible to participate in primary and secondary outcome components regardless of whether they have an unpaid carer who gives informed consent to take part.

3.2.2 Unpaid carers

1) Be identified by a patient who has given verbal informed consent to participate in the study as providing them with substantial emotional and practical support in an unpaid capacity;
2) Provide verbal informed consent;
3) Have spoken and written English proficiency sufficient to complete the Carer Experience Scale (CES) (3) and/or interview.

3.2.3 Centre staff

1) Be employed on a permanent basis either full- or part-time at a participating cancer or palliative care centre in a role that provides clinical care to patients with cancer pain or front desk administrative support;
2) Provide written informed consent.

3.3 Exclusion criteria

3.3.1 Patients

- Participation at another centre taking part in the trial or at the same centre at a time when it was in the opposing arm;
- Documented as having cognitive impairment that would preclude capacity to give informed consent.

3.3.2 Unpaid carers

- Patient for whom they provide care is not participating in any secondary outcome components.

3.3.3 Centre staff

- Working at a participating centre on a casual or agency basis.

INVESTIGATIONAL PLAN

3.4 Study design

A stepped-wedge cluster randomised controlled trial with oncology and palliative care centres as units of randomisation will be undertaken to evaluate implementation of the cancer pain guidelines.

In this design, randomisation concerns the sequence in which the intervention begins at each centre rather than whether it occurs at all (see Figure 1).

Centres	Phases		
1	Control	Training & Intervention	Sustainability
2	Control	Training & Intervention	Sustainability
3	Control	Training & Intervention	Sustainability
4	Control	Training & Intervention	Sustainability
5	Control	Training & Intervention	Sustainability
6	Control	Training & Intervention	Sustainability
7	Control	Training & Intervention	Sustainability
8	Control	Training & Intervention	Sustainability

3.4.1 Figure 1. Stepped wedge design with staggered introduction of training/intervention in 8 centres

3.5 Rationale for study design

While needing a slightly longer timeline than a parallel design, a stepped-wedge cluster randomised controlled trial has the following translational research advantages:

1) all centres will receive the intervention so have an added incentive to participate;
2) the design controls for between-centre variation in baseline practice;
3) statistical power is boosted by the opportunity to assess intervention effects in a pre/post comparison across centres;
4) at any given time beyond the baseline period, the design allows a randomised comparison of intervention and control centres; and
5) assessment of longitudinal effects (e.g. sustainability) can occur (42).

A stepped-wedge cluster randomised controlled trial design also allows greater diversity of centres to be included to allow broad applicability of the results, without compromising comparability or requiring matching of sites. Recruitment during the control arm at each centre will commence at least 3 months prior to training to enable sufficient time for the smaller centres to recruit the target sample.

Outcome data collection for patients in the control arm will be censored prior to their centre making the transition to the intervention arm. For a period of 4 weeks after the training phase, data will not contribute to outcome measurement, to allow time for the intervention to become consolidated as routine practice.

Importantly, patients can participate in only one of the arms, not both. This means that patients who participate at their centre during the control arm will be ineligible to participate in the intervention arm.

3.6 Randomisation

Randomisation will be concerned with the time at which each centre transitions from the control arm to training and intervention. Stratification will be unnecessary because each centre will serve as its own control. Patients will be automatically allocated to usual care or intervention arms according to the current arm of their treatment centre upon their first presentation. Participants will be sequentially allocated an ID number, which will be used for all subsequent study documentation for that participant.

3.7 Blinding

The nature of the intervention in this study renders blinding of health professionals impractical. Information and consent forms for patients will provide only general information about the aims of the study (i.e. that it will compare different approaches to cancer pain management) rather than the specifics of the design and intervention (see Appendix 2). Health professionals will be asked not to discuss the study design or current arm at their centre with patients and carers.

Previous experience of cluster RCTs by the current team also suggests that attempts to blind research assistants collecting data will be impractical. Instead, attention will be paid to research assistant training and standardization of data collection as ways to limit the potential for bias.

Personnel conducting analyses will be blinded to centre allocation.

4.0 Control and intervention arms

4.1 Description

Each participating centre will first be in the control arm and then transition to the intervention arm, defined as follows.

4.1.1 Control arm

Management, staff and patients at participating centres will be made aware of the Australian guidelines on the Cancer Council Australia Cancer Guideline Wiki and the study's aim to test implementation of these. Where an existing screening system is not already in place, centres will be equipped with a system for symptom screening and providing reports for treating clinicians to consider during the consultation. Screening measures will be made available in Chinese, Italian, Greek, Vietnamese and Arabic, which are the languages spoken in Australia most commonly associated with poor English proficiency (43). Centres will otherwise provide care to patients with cancer pain according to local practice and will not be given training or strategies to support implementation of the screening system or guidelines.

4.1.2 Intervention arm

At the beginning of a ***training phase***, two 'clinical change champions' will be identified at each site to take a leading role in administering the intervention with support from the project team. Two are needed to cover for periods of absence by one of the champions. Selection of clinical champions will be conducted in partnership with managers at each centre. Champions can be of any discipline and role provided they meet the criteria summarised in Box 1.

**Box 1. Features of organisational change champions identified by Shaw et al (2012) (44)**
• Has authority to cultivate an environment for ongoing practice improvement/organisation learning<br>• Has a clear vision for the larger organisation and effectively communicates how the project-based innovation fits into that vision<br>• Ongoing role<br>• Actively and enthusiastically promotes both the specific project as well as ongoing practice improvement<br>• Mobilizes resources (internal/external) for ongoing practice improvement<br>• Navigates the socio-political environment for ongoing practice improvement<br>• Provides leadership for ongoing practice improvement

Change champions will attend 2-day training at their own site aimed at tailoring the intervention to local needs and contexts. Champions will be supported and trained to administer the audit tool to identify strengths and weaknesses at their centre against recommendations in the guidelines. They will also be trained to identify barriers and facilitators to cancer pain management and helped to develop solutions. They will be trained in the use and trouble-shooting of the pain screening system. Finally, they will be trained to provide information to patients and train them in using the patient-held resources, as well as to induct new staff into use of the guidelines at their centre.

Also during the training period, all clinical and front of house administrative staff will attend a half-day workshop at their centre in which they will be given an overview of the guidelines, summary clinical pathway and implementation resources by the change champions at their site with support from the project team. Medical and nursing teams will undergo online training in pain assessment

and management via QStream modules to assess their understanding of recommendations in the guidelines.

During the ***intervention phase***, the process for feeding back individual patient results from screening will be monitored, with medical teams receiving a summary report of screening data for each patient prior to consultation to inform management, and a copy filed in the medical record. The process by which this happens will vary by centre and can be electronic (e.g. email) or hard copy (automatic print-out, carried into the consultation by the patient or administrative staff). Adherence to standards of pain screening, assessment and management will be monitored via the audit tool. Feedback will be at the centre level rather than refer to any individuals, and data will be recorded and fed back in such a way that no individual can be identified. Health professionals selected to undertake audits will do so under the supervision of a project team member until the latter is satisfied they are able to conduct audits to the standard required. Future audits will be observed periodically throughout the study. Health professionals conducting the audits will be invited to engage in open communication with the project team at any time. Patients will be provided with patient-held resources and brief (5 minute) training on how to use them. Resources will include a pain diary, personal goal setting support, a tool for monitoring capacity to self-manage pain and identify related needs and a pain management plan detailing medications and out-of-hours contacts. The degree to which champions take on all responsibilities or delegate these to other staff who they train in carrying out relevant tasks will be tailored to the individual site. Those rolling out the intervention at each centre will be invited to participate in regular teleconferences with staff at similar sites and members of the project team to share learning and solve problems.

During a ***sustainability phase***, site specific health professionals who are trained in the use of the audit tool will provide the research team with de-identified audit feedback data on patients screened as having a severe pain score ≥7 NRS to assess whether adherence to standards of pain screening, assessment and management have continued.

4.2 Concurrent treatments

Trial patients will continue their usual treatment regimen in all respects other than those prescribed for cancer pain when in the intervention period.

4.3 Post study treatments

All participants will be followed by their clinician for continuing care, irrespective of the point at which they exit the study.

5.0 Study procedures

5.1 Identifying and recruiting participants during intervention period

5.1.1Patients

5.1.1.1 Identification of potential participants

All patients screening ≥2 NRS for worst pain of any cause at oncology centres who have current active cancer as identified by treating clinicians will be eligible for inclusion in components of the study examining secondary outcomes, although only patients screened with worst pain ≥5 (moderate-severe) will have data included in the primary analysis. Eligibility for components of the study focusing on secondary outcomes will be screened by a research assistant against other criteria on initial approach.

The screening form will give patients an opportunity to opt out if they do not wish to be contacted (see Appendix 2 for wording). The project team will extract contact details for patients scoring ≥2 on a 0-10 NRS who do not opt out. All participants who score worst pain ≥5 on a 0-10 NRS who do not opt out will be contacted either in person at the centre or via telephone and invited to give verbal consent to rate their pain a week later for the primary outcome. Where screening data indicates that the patient chose to complete measures in a language other than English, healthcare interpreters will be employed to translate the verbal consent and data collection exchange between the research assistant and the patient. (Appendix3). Additionally, recruitment to research aimed at answering secondary objectives of the project relating to pain, empowerment, QOL and cost effectiveness and the qualitative sub-study will occur by approaching patients who score 2 or more on a 0-10 NRS at the centre or by telephone. Where patients cannot be approached at the centre, the team will telephone the patients to ask if they are interested in learning more about the study and seek their informed consent to participate. A cover letter, two copies of the participant information and consent forms will be posted out together with copies of the questionnaires and a reply-paid envelope. See Figure 2 and 3 for flow diagrams of recruitment of patients to primary and secondary outcome components respectively.

A Participant Master Index (an excel file to track participant ID numbers and progress through the study) will be kept of all patients who screen ≥2 NRS for worst pain including the reasons for non-entry regarding failure to meet inclusion criteria and refusal.

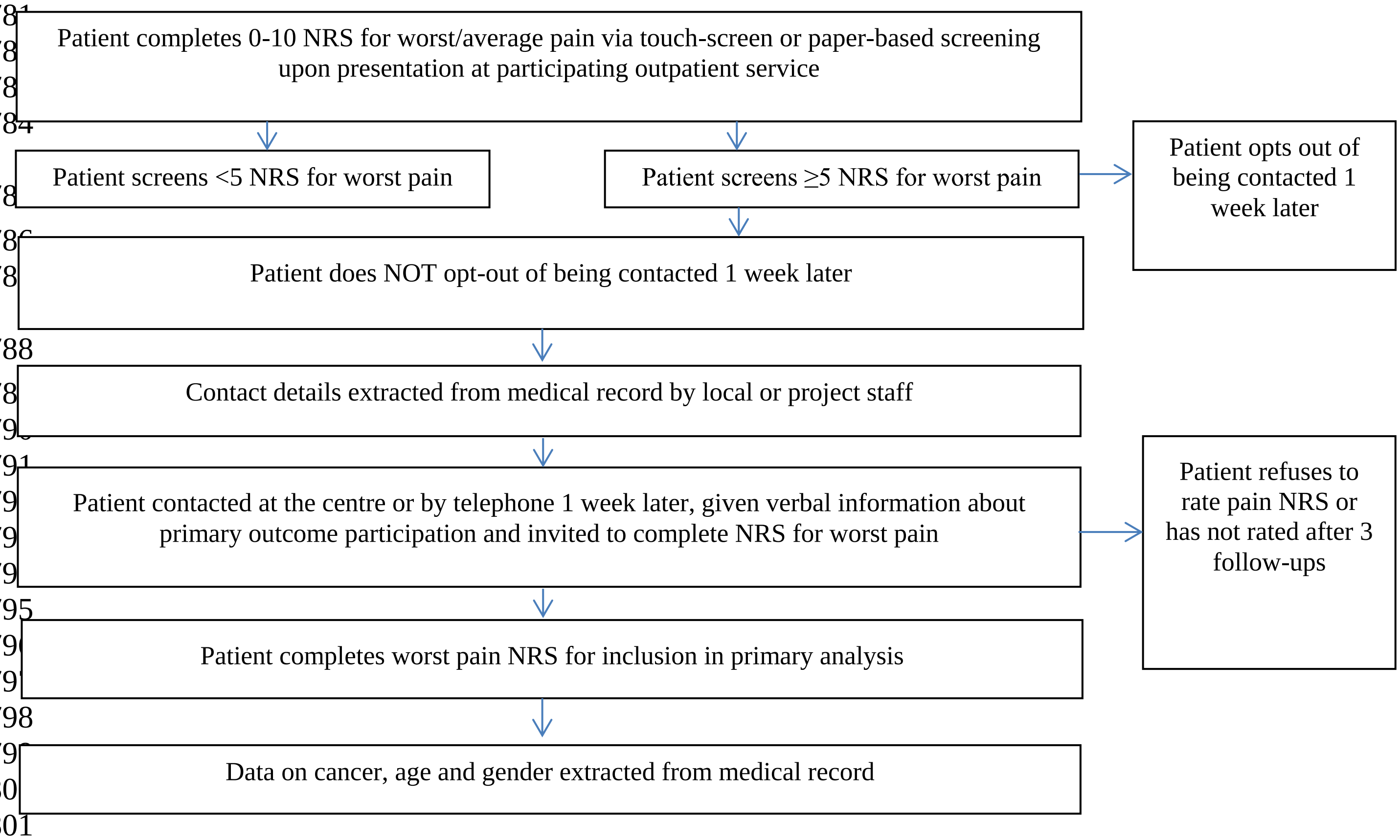

NOTE: The above flow in patient recruitment and data collection occurs regardless of whether the centre is in the control or intervention arm.

5.1.2 Figure 2: Study diagram of patient recruitment and data collection for primary endpoint of worst pain at 1 week after positive screen

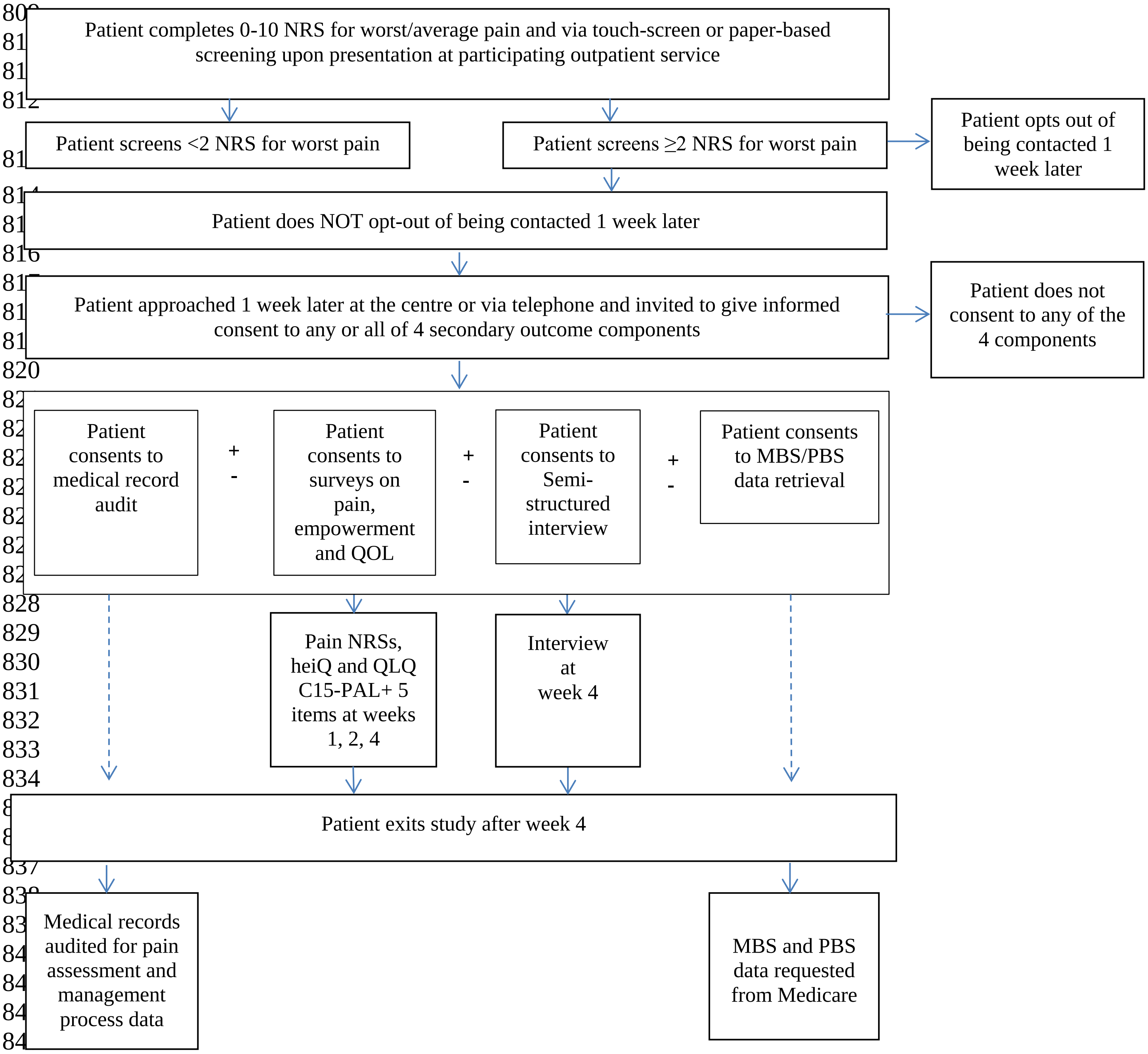

5.1.2 Figure 3: Study diagram of patient and carer recruitment and data collection for secondary outcomes

NOTE: The above flow in patient recruitment and data collection occurs regardless of whether the centre is in the control or intervention arm.

ABBREVIATIONS: EORTC QLQ C15-PAL = European Organisation for the Research and Treatment of Cancer Quality of Life Questionnaire C15-Palliative care; MBS = Medicare Benefits Scheme; NRS = numerical rating scale; PBS = Pharmaceutical Benefits Scheme

5.1.2.1 Consent process

This study will use four procedures relating to consent:

1. A <u>waiver on patient consent</u> to use de-identified screening and audit data for endpoints comparing average pain and adherence to key standards of cancer pain assessment and management within the patient population during intervention and sustainability versus control periods;
2. An <u>opt-out procedure</u> for patient permission to obtain their contact details to telephone them 1 week later about the research for the primary outcome of worst pain on a 0-10 NRS 1 week after a positive screen. Upon contact, patients will be invited to give verbal consent to provide primary outcome data but will still be at liberty to refuse at that time;
3. A verbal informed consent procedure for secondary outcome data (surveys, interviews and medical record audit);
4. A <u>written informed consent procedure</u> for involvement by patients in the study's health economic evaluation.

The National Health and Medical Research Council (NHMRC) National Statement on Ethical Conduct of Research (2007) includes provision for waiving and an opt-out approach to consent in Chapter 2.3 (45).

5.1.2.2 Waivered consent

A waiver on consent will be sought to use pain screening data to compare patient populations between centres at baseline and to see whether this population is changed in terms of pain by the intervention. As well as screening data, a waiver on consent will be requested to collect data on age, gender and cancer type to contextualise screening data, compare patient characteristics between centres and establish the representativeness of samples for primary and secondary outcome assessment. These data will be extracted by local staff or project staff. Screening measure data will be entered onto an Excel spread-sheet by local or project staff. For patients who score $\geq$2 and who do not opt out, their name and contact details will be entered onto the spread-sheet. Names and contact details for those patients who score NRS 0-1 for worst pain or who opt out of being contacted will not be entered onto the spread-sheet.

5.1.2.3 Opt-out procedure

In the opt-out procedure, patients will indicate consent to be contacted 1 week after screening positive for a pain NRS to be administered if they do not tick a box of the screening survey to request that this should not occur. The screening form will include information about the project and that patients will be contacted at the centre or via telephone 1 week later about their pain if they do not tick the box. When the contact is made, the researcher will identify who they are, explain why they are contacting the patient and the purpose of this information, together with reassurance regarding confidentiality to ensure the participant still wishes to continue (Appendix 3). Patients will be given the opportunity to provide verbal consent to provide primary outcome data but will still have the option of refusal at that time. The opt-out procedure has been chosen because, when evaluating a health system level intervention, selection bias in the primary outcome would substantially impact on the ability to interpret the true efficacy of the intervention. The data being requested of participants is minimal and poses minimal risk to the participants. Where patients report pain $\geq$2 at this follow-up contact, they will be provided with contact details for their medical team to follow up if they so wish. Patients will be deemed lost to follow-up if they have not

provided pain NRS data of been given an alternative day/time to make contact after 3 attempts to contact on 3 consecutive days.

5.1.2.3 Verbal consent procedure

Patients will be invited to give informed verbal consent to provide information for primary and secondary outcome analysis. These components will include the following:

1. Follow-up surveys: consent to be contacted at the centre or via telephone at intervals of 1, 2 and 4 weeks to answer two questions about pain, 15 questions about quality of life and 19 questions about sense of control over pain management;
2. A semi-structured interview: consent to be interviewed for up to 30 minutes about satisfaction with care for my pain and any information and resources they have been given by the service they attend.
3. Access to medical records: consent for the research team to access medical records relating to how pain has been assessed and managed and any hospitalisations they may have during my time on the study;

A researcher will use scripted wording to ensure patients' consent is informed and check understanding (Appendix 3). Verbal consent will be audio-recorded and kept on file as a record.

Obtaining informed verbal consent for secondary outcome evaluation in this study will be a process of information exchange between the study staff, the potential participant and any other person the potential participant believes should be included in the discussion. The participant information sheet will be used as a basis for the discussion, which will cover all procedures, benefits and burdens expected of possible during the study. The participant will be given opportunity (in time and physical capacity) to consider the study and formulate questions. Any questions will be addressed and answered fully.

Prior to study commencement, the research assistants will be trained in consent procedures for this study, with the opportunity to role play scenarios to ensure all information is fully covered. The consent form will be completed by the participant verbally rather than in writing.

.

Participants will keep a copy of the PISCF for their records.

5.1.2.4 Written informed consent

In addition, patients will be given the opportunity to sign a separate, dedicated Medicare consent form giving permission for MBS and PBS data regarding their healthcare utilisation to be included in a component of the study evaluating cost-effectiveness.

5.1.3 Unpaid Carers

5.1.3.1 Identification of potential participants

Patients who provide verbal informed consent to take part in one or more secondary outcome components will be asked whether they have an unpaid carer who meets eligibility criteria and might be interested in taking part. Where the answer is positive, the name, address and telephone number of the carer will be recorded and a letter and two copies of a carer-specific PISCF and reply-paid envelope posted out to the carer/or given to the participant to give to the carer and then followed up by telephone or at the centre.

5.1.3.2 Screening for eligibility

Screening of carers will be undertaken by research assistants via discussion with the carer themselves.

5.1.3.3 Consent process

Consent will follow the same verbal consent procedure as for patients. Carers can give consent to participate in either or both of the following components:

1. Assessment of carer experience via survey;
2. Qualitative sub-study involving a semi-structured interview.

5.1.4 Centre staff

5.1.4.1 Identification of potential participants

The majority of staff at each site will be eligible to participate. Staff will primarily be invited to participate via email circulars. The team will also raise staff awareness of the project and invite staff to participate by presenting at staff meetings.

5.1.4.2 Consent process

A waiver on consent will be requested from the HREC to send out an email circular to staff with a Survey Monkey link (Appendix 4), inviting them to provide anonymous information about the time they have spent on cancer pain screening, assessment and management on their most recent full work day (Appendix 5). Completion of the survey will be taken as evidence of informed consent. Staff will be approached to take part in focus groups/interviews via the best local means, as advised by centre managers. It is envisaged that these means may include email circulars (see Appendix 6), leaving PISCFs in pigeon-holes and/or in the staff room as well as direct approach by a project assistant. Information about the study will include a contact number for staff to ring if they have any questions, and project staff will visit each site regularly giving staff the opportunity to approach in person. A secure ballot-style box will be left in staff rooms for staff who consent to return their completed consent forms between project team visits. Where staff are approached in person, the project assistant will begin by canvassing initial interest and, where necessary, arranging a later time to undertake the informed consent procedure at the staff member’s convenience to minimize interference to work.

5.1.5 Withdrawal

Participants of all kinds will be free to withdraw at any time without jeopardizing their relationship with the centre or researchers. Whilst participants are not obliged to give a reason for withdrawal, where this is volunteered, the reason will be recorded so that any consistent reasons can be identified and addressed by changes to the protocol.

5.2 OUTCOMES AND MEASURES

The study measures are tabulated in Table 1. The study period for each patient will be 4 weeks or until death whichever is the shorter time. Validated measures are reproduced in Appendix 7.

5.2.1 Primary outcome

The primary outcome will be the probability that patients screened as having moderate-severe (≥5) worst pain will experience a clinically important improvement 1 week later, as measured by 30% on a 0-10 numerical rating scale (NRS).

Rationale:

- One week following positive screen is considered sufficient time to allow comprehensive assessments to have been conducted and any new treatment regimens to have become established.
- The past 24 hours has been chosen as a recall period balancing the need to avoid recall bias whilst sampling a representative time window.
- The NRS is the optimal brief measure of pain severity on the basis of compliance rates, responsiveness, ease of use and applicability (46). The NRS is also the preferred response option of the most widely used and validated multi-dimensional pain scales (e.g. Brief Pain Inventory (47)).
- A threshold of 5 on the 0-10 NRS has become established for moderate cancer pain (48), and patients with pain of this magnitude form the focus of the primary endpoint because of the added urgency of reducing pain when it is moderate to severe versus mild.
- Thirty percent has been selected based on recommendations for pain trials by the US Food and Drug Administration and takes into account research evidence that the number of points needed for a minimally important difference (MID) varies according to the baseline score (49).

5.2.2 Table 1: Patient and carer measures

	Screening	Following consent	Weeks 1, 2 & 4	Exit (after Week 4)
Medical file review				
DOB		*		
Gender		*		
Language spoken at home		*		
Aboriginal or Torres Strait Islander status		*		
Diagnoses (cancer and other)		*		
AKPS/ECOG				*
Analgesic use				*
Patient measures				
Pain NRS	*		*	
EORTC QLQ-C15 Pal + 5 items			*	
heiQ			*	
Availability of primary carer		*		
Pain assessment and management audit				*
Carer measure				
CES			Wks 2 & 4	
Medicare data				*

AKPS = Australian Karnofsky Performance Scale; CES = Carer Experience Scale; DOB = date of birth; ECOG = Eastern Cooperative Oncology Group Performance Status; EORTC QLQ-C15 Pal = European Organisation for the Research and Treatment of Cancer Quality of Life Questionnaire - Palliative Care; heiQ = Health Education Impact Questionnaire; NRS = numerical rating scale

5.2.3 Secondary outcomes

5.2.3.1 Patient pain

Secondary outcomes will relate to the potential for the intervention versus usual care arm to result in the following:

1. Increase in mean reduction for worst pain severity in patients with clinically significant (≥2) and moderate-severe (≥5) worst pain from screening to 1 week later by half a standard deviation (0.5 SD). A MID will be assumed to be 0.5 standard deviation (SD), which is an established rule of thumb for patient reported outcomes (50).
2. Decrease in mean scores for worst and average pain across all screenings to give a population measure of effect. As for the primary outcome, the MID will be considered to be a 0.5 SD difference between control and intervention arms.

5.2.3.2 Patient quality of life

Patient QOL will be measured at 1, 2 and 4 weeks using the EORTC QLQ C15-PAL, a short-form of the EORTC QLQ-C30, which is perhaps the most widely used cancer-specific QOL measure (1, 51). The QLQ C15-PAL includes 15 items to reduce burden and focus on issues most relevant to people with advanced cancer. It provides scores for global QOL, physical functioning, psychological functioning, and symptoms that include fatigue, nausea and vomiting, dyspnea, insomnia, appetite loss and constipation. Importantly for this study, it also provides a score for pain interference and uses a recall period of the past week - both features will complement the pain NRSs which focus on pain severity over the past 24 hours. In this study, 5 additional items from the QLQ-C30 will be added to the C15-PAL at every administration to enable cost-utility evaluation using the the QLQ-Utility measure (53) (see below). These items assess physical functioning, role functioning, social functioning (2 items) and diarrhea, and thus also provide useful information for clinical management.

As for pain outcomes, the MID will be considered to be a 0.5 SD difference between control and intervention arms. This offers a convenient single estimate that approximates to scale-specific MIDs for deterioration and improvement developed for the QLQ C15-PAL (52).

5.2.3.3 Patient empowerment

The Health Education Impact Questionnaire (heiQ) will be administered at weeks 1, 2 and 4 to evaluate patient empowerment. A version of the heiQ developed in Australia specifically for cancer will be used that includes scales measuring Health service navigation, Constructive attitudes and approaches and Skill and technique acquisition (2). As well as offering an index of self-reported self-management efficacy, it is hypothesised that patient empowerment may have a direct impact on pain experience itself (29).

5.2.3.4 Carer experience

The Carer Experience Scale (CES) will be administered at weeks 2 and 4 to evaluate the intervention effect on unpaid carers in terms of their relationship with patients, support received and ability to carry on with their own lives (3). As for pain and QOL outcomes, the MID will be considered to be a 0.5 SD difference between control and intervention arms.

5.2.3.5 Cost-effectiveness

A cost-effectiveness analysis will evaluate the incremental costs and consequences of the implementation strategies relative to usual care. Patient outcomes will include response at the end of Week 1 (response is defined as a clinically important improvement of 30% on a 0-10 NRS 1 week post-screening for those with moderate-severe (≥5) worst pain) for the primary cost-effectiveness analysis and quality adjusted life years (QALYs) based on utility scores from the EORTC QLQ-C15-PAL and 5 additional items from the QLQ-C30 (mapped from QLQ-Utility (53)) and index values for carers using the CES for a modelled cost-utility analysis. Survey responses will be linked to health service utilisation data accessed through the Centre for Health Record Linkage and from Commonwealth datasets (Medicare; PBS) and hospital records. Variables will include: ARDRG code; number of hospitalisations; length of hospital stays; number of emergency department, outpatient, psychology/psychiatry and GP visits; health insurance status; medication usage. Costs of the intervention will relate to the screening systems implemented at each centre, materials for training and the manual, and staff time spent on attending training and screening, assessing and managing cancer pain (see staff-level process measures below).

5.2.4 Structural and process measures

The implementation strategies evaluated by this study represent a complex intervention involving multiple interacting components (41). As such, measures of infrastructure and processes for care are needed to assess fidelity of implementation, clarify causal mechanisms and identify influential contextual factors to inform further refinement, adaptation and tailoring to different settings.

5.2.4.1 Centre-level structural measures

Centre staffing, equipment and access to specialist services, including staff turnover as a variable that may influence the sustainability of implementation.

5.2.4.2 Centre-level process measures

Staff training (via QStream and other initiatives), implementation of and adherence to the guidelines and documentation quality. Discussion of pain and other symptoms and their management at multidisciplinary team meetings/case reviews will be tracked.

5.2.4.3 Staff-level process measures

Time spent in training and on cancer pain screening, assessment and management by clinical staff and front desk administrative staff. Staff will be invited to complete an anonymous online survey via the Survey Monkey platform that asks them their role at the centre and three separate questions on how much time they spent on cancer pain screening, assessment and management during their last complete work day (Appendix 5).

5.2.4.4 Patient-level process measures

Number and duration of consultations, multi-disciplinary input, primary care support, and standards of care recommended by the guidelines. Items of particular interest will be instances of screening, assessment, patient education, regular analgesia (agent, route, dose), breakthrough analgesia (agent, route, dose, and number of times taken) and management of side-effects (preventative and treatment), as well as any other anti-cancer, interventional and non-pharmacological methods used to address pain. The clinical audit tool (administered by research assistants to standardise between conditions/centres), will assess participants' clinical records retrospectively. Because it is often not documented in medical records, patients' receipt (or otherwise) of pain education will be measured by asking the patient at each time-point for telephone follow-up.

5.2.5 Descriptive/control variables

5.2.5.1 Patients

5.2.1.1.1 Demographics

1. Age
2. Gender
3. Availability of an unpaid carer:
    a. lives with carer
    b. lives alone but carer available
    c. lives with non-carer
    d. lives alone, no carer
4. Postcode
5. Country of birth
6. Language spoken at home
7. Aboriginal and/or Torres Strait Islander status

5.2.1.1.2 Cancer diagnoses

The following clinical data will be collected:

i. Site
ii. Tumour stage
iii. Sites of metastases
iv. Time since diagnosis
v. Current treatment

5.2.1.1.3 Comorbidities

Comorbidities will be identified that influence pain or its management according to the guidelines. Conditions of interest will include painful conditions (e.g. arthritis), renal impairment, gastro-intestinal ulcers and conditions causing cognitive impairment (e.g. dementia).

5.2.1.1.4 Performance status

Performance status will be measured using whatever tool is routinely in practice at each site. It is anticipated that these will most often be the Karnofsky Performance Status (KPS) (54) or Australia - modified Karnofsky Performance Status (AKPS) (55) at palliative care sites and the Eastern Oncology Cooperative Group (ECOG) Performance Status (56) at oncology sites. Conversion between these scales to enable comparability will be undertaken via validated methods where available (57).

5.2.1.2 Carers

1. Age
2. Gender
3. Relationship to patient (child, parent, partner, other)
4. Lives/does not live in the same household as the patient
5. Country of birth
6. Language spoken at home
7. Aboriginal / Torres Strait Islander status

5.2.1.3 Centre staff

Role (front desk administrative, medical, nursing, allied health).

5.3 Qualitative sub-study

Patients and carers (n=20 respectively) who participate will be invited to give informed consent to take part in a qualitative sub-study. Interviews will focus on experience and usefulness of symptom assessment and management, the patient-held resources and degree of person-centredness and coordination of care using a focussed question route. See Appendix 8 for an interview guide.

Health professionals who give informed consent will participate in staff focus groups or interviews at each centre which will explore perceptions of what has worked well regarding the implementation strategies and potential improvements. See Appendix 9 for a focus group/interview guide.

5.4 SAFETY MONITORING

5.4.1 Reporting of incidents

Clinical staff and managers at participating centres will be encouraged to report to the project team any adverse incidents that they believe may be related to the project intervention or study procedures. See Appendix 10 for the template.

5.4.1.1 Follow-up of incidents

After the initial report, investigators are required to follow-up each incident and provide further information both to the coordinating centre (ImPaCCT office, UTS) and the approving HREC if required. All incidents reported as ongoing are to be reviewed at subsequent visits or appointments in order to report progress and resolution.

All events are to be followed until:

- resolution;
- the event can be explained;
- a participant involved is lost to follow-up;
- a participant involved dies.

Reports are to contain details of follow-up investigations, result reports or reports from other consultations, and are to be updated in a report to the coordinating centre and the approving HREC.

5.4.2 Stopping rules

The study will be stopped if or if reporting of incidents indicate that review of the study protocol is required or if new literature indicates findings that answer the research questions.

5.4.3 Monitoring

Reports of incidents will be sent to the Trial Management Committee and the approving Hospital Ethics Review Committee within two weeks of knowledge of the event.

8.1.1.1 The Trial Management Committee

Each meeting of the Trial Management Committee will receive from the coordinating centre a summary report of the incidents reported by the investigators. This summary report will be reviewed for reporting compliance, trends in incidents, and outstanding incidents that require specific attention. All Trial Management Committee discussions will be minuted, with actions detailed, and reviewed at the subsequent meeting.

8.1.1.2 Hospital Ethics Review Committees

Incidents that meet the requirements to reported to the approving HREC will be reported in the required format and timeframe stipulated by each individual committee.

5.5 STATISTICS

5.5.1 Statistical analysis

For the main analysis, linear mixed models (5) will be used to model the outcomes of interest, while accounting for the clustering and longitudinal design. Although designed for the analysis of continuous outcomes, we have conducted computer simulations to confirm that this approach will work very well for the pain and other NRS scores that are measured on a ten point scale. The linear mixed modeling framework is very flexible. For example, it will allow for testing whether treatment effects diminish over time. It will also allow the incorporate of additional covariates of interest, for instance patient age, gender, ethnicity and other factors related to their type of disease; and inclusion of covariates that reflect characteristics of the study centres. Statistical analysis will be performed using the library lme4 in the R package (58). Analyses will be repeated for all patients and patients newly referred during the intervention arm to control for the possibility that outcomes are influenced by care received prior to the intervention being implemented. For secondary patient outcomes, analyses will be repeated for patients with clinically significant (≥2) and moderate-severe (≥5) worst and average pain.

5.5.2 Sample size and power calculations

To assess the appropriate study sample size and compute study power, we used a carefully designed computer simulation allowing for 20% dropout by both sites and patients. Drawing on data from 1,612 consecutive patients (59), outcome data were generated to mimic pain scores we expect to see in the presenting population. We generated data from a beta distribution (scaled 0 to 1), rescaled to lie between 0 and 10, then rounded to integer values. We then discarded patients with scores of <2 to represent the sample of interest. We allowed centre-specific means to vary between .05 and .15 to generate an appropriate intra-class correlation. We used a standard minimally important difference of .5 effect size (60). To more closely represent reality, we allowed for variation in the responses of each patient to intervention. Once a hypothetical study population had been generated, we ran a linear mixed model that included an intervention effect as well as an intervention by time effect, as well as a random centre effect. We repeated this whole process several hundred times and then estimated power by computing the proportion of times that the null hypothesis would be rejected. We did this both for the main intervention effect, as well as the intervention by time effect. Two-sided hypothesis tests with type I errors of 0.05 were used. Assuming 82 patients per centre at 6 sites (N=492), our study will have >90% power to detect the main effect. The study will oversample by 2 sites (i.e. a total of 8 sites) and 18 patients per site (i.e. N=100 at each site) to allow for drop-out.

5.5.3 Cost-effectiveness analysis

The primary outcome for cost-effectiveness analysis will be the incremental cost per additional responder at the end of Week 1 (response is defined as a clinically important improvement of 30% on a 0-10 NRS 1 week post-screening for those with moderate-severe (≥5) worst pain). Quality of life data collected at weeks 1, 2 and 4 will be used to conduct a modelled cost-utility analysis. As economic data may be skewed, confidence intervals will be estimated with bootstrap methods (6). Sensitivity analysis will examine the effect of assumptions and determine which cost components drive the results.

5.5.4 Qualitative analysis

Thematic coding and classifying techniques will underpin the analysis of semi-structured interviews and focus groups (7).

6.0 ETHICS

6.1 Benefit anticipated from the study

The proposed project aims to test cost-effectiveness of an intervention with potential to enable equitable care for people with cancer pain across Australia. Currently, patients are subjected to a 'lottery' regarding quality of care, especially in regional and rural areas. While this is partly due to lack of specialist services, research suggests that even the basics of pain assessment and management are often not offered, despite making limited requirements on resources. The guidelines to be implemented in this project include recommendations and processes to ensure minimum standards of pain assessment and management can be achieved regardless of the availability of specialist services. Another key to the problem is empowering patients and carers to self-manage pain and take the lead in coordinating their care with appropriate resources and support. The implementation package to be tested in the proposed project includes a patient-held resource to help patients advocate for person-centred, evidence-based care.

Subgroups of people with advanced breast and other cancers who stand to benefit disproportionately from intervention are patients with limited English and health literacy. Australian research suggests cancer patients from non-English speaking backgrounds may have greater unmet needs arising from problems with communicating with health professionals and lack of knowledge of the health system. In the proposed project, tools for pain screening and patient-held resources will be translated into community languages to ensure that medical teams are made aware when patients have pain without relying on verbal communication. Patient-held resources have been developed for a basic level of literacy and are intended to empower patients to manage their pain and support discussion with medical teams regarding pain management plans and who to contact for support under different circumstances.

6.2 The possibility of psychological stress

Some participants may experience stress associated with recounting their experiences when completing interviews for the qualitative sub-study of this project. This is a vulnerable population, where sensitive issues about ability to function and other questions may raise broader issues of psychological distress.

Participants who take part in interviews will have chosen to do this as an 'optional extra' in addition to participation in the rest of the project. There will be no deception of participants at any stage. Each participant interaction will be undertaken by carefully selected and trained study staff. This training will initially be undertaken in conjunction with investigators and senior research personnel, who have been trained in Good Clinical Practice, to ensure that staff are able to detect and monitor participant distress.

Although there may be acute distress, the weight of evidence is that such 'prompt' questions are in fact an avenue to open up discussions which are well regarded by people despite their initial potential distress.

6.3 Research on people in dependent relationships

The nature of doctor-participant relationships dictates that patient and carer participants may feel that they are in the dependent position. The investigators and their designees will work to minimise any concern of inappropriate influence—the presentation of the study will be as unbiased as possible, the information sheet and consent forms will be clear, and participants will be able to withdraw from the study at any time. A person not directly involved in the clinical care of the participant will collect primary outcome data and obtain consent to provide data for secondary outcomes. Research assistants undertaking informed consent will be trained to identify their roles as research-only and to present the study in such a way that clinical care is separated. Training will provide an opportunity for project staff to determine appropriate ways of dealing with clinical situations that might arise during their research visits. Patient participants will be cared for as individuals with specific needs; the needs of research will come second.

Where patients report pain of NRS 2+ to research staff at follow-up, research staff will provide them with contact details for their medical team and invite them to call for assistance. It is not the research staff's role to provide clinical care to patients.

6.4 Method and nature of recruitment and advertising

Participants will be recruited following initial screening in the participating clinics at each site. Any participant who is approached to take part in this study has the right of refusal. Refusal to take part in this study will not adversely affect the provision or quality of care provided to any participant in any way.

6.5 Protection of privacy and preservation of confidentiality

All participants will be allocated a unique ID number. The master list linking identifying participant information and ID number will be maintained in a locked cabinet, separate from the participant database. Form tracking will be via participant ID number only. There will be master lists held at each participating site and at the coordinating site. The participant database will be stored on a password-protected hard drive maintained by the study investigators. Data will be analysed by ID number only.

Data relating to the primary outcome (screening, cancer and other diagnoses, age, gender and language spoken at home) will be de-identified before leaving each centre for those patients who do not consent to take part in secondary outcome assessments.

6.6 Restriction of use of data

Investigators will have access to data by ID number only for the purposes of data monitoring and analysis. The Project Coordinator will have access to all study data for the purposes of data checking, monitoring and preparation for analysis. Study research assistants and site investigators will have access to the local site consent forms, Case Report Forms and the data contained within for the purposes of data collection, data entry and data query resolution. Study auditors will have access to Case Report Forms (by ID number only) and study files in order to audit the study. Site research ethics committees will have access to local data for audit purposes.

6.7 Use of personal information

Only enough personal information to give a general demographic and disease profile of the participant will be collected, along with details needed to enable contact via post and telephone. Data on language spoken at home will be collected because of the hypothesis that patients who are

less proficient in English will benefit especially from the intervention. The participant responses collected are limited to those that will address the study's primary and secondary aims.

6.8 Estimated time of retention of personal information and planned disposal

Records from the study will be maintained for 15 years after study completion in secure archiving facilities. Once the 15 year waiting period is complete, the files will be erased from the database hard-drive and any paper copies shredded, including the master list linking participant name and treatment number.

The data will be retained in accordance with good clinical practice recommended by the NHMRC National Statement and the GCP guidelines, and in a form that is at least as secure as the sources from which it was obtained.

7.0 STUDY ADMINISTRATION

7.1 Data handling and record keeping

7.1.1 Direct access to source data

A statement of permission to access source data for regulatory and audit purposes will be included within the PISCF with explicit explanation about this given as part of the consent process. Specifically, access will be required by study staff (including investigators, site coordinators and study nurses), Hospital Research Ethics Committees (HRECs) and the data management team. In addition, de-identified data will be made available for meta-analysis and where requested by journals for publication purposes.

Case Report Forms (CRFs) for patients and carers will include:

- CRF – Patient Primary Data (week 1)
- CRF – Patient Secondary Data (weeks 1, 2, 4)
- CRF – Carer Data (weeks 2, 4)

7.1.2 Data collection

Data will be sourced from the following.

Measure	Source	Completed by:
Efficacy on patient pain	Screening data / CRF	Automated / research assistant / local trials nurses
Efficacy on patient QOL	CRF	Automated / research assistant/ local trials nurses
Efficacy on patient empowerment	CRF	Research assistant/ local trials nurses
Performance status	Medical records	Research assistant/ local trials nurses
Efficacy on carer experience	CRF	Research assistant/ local trials nurses
Resource utilization (individual)	MBS/PBS data	Research assistant/ local trials nurses
Semi-structured interview / focus groups	Recordings	Research assistant

Structural data – centre level	Audit	Research assistant/ local trials nurses
Structural data – staff level (staff time)	Online survey	Centre staff
Process data – patient level	Audit	Research assistant/ local trials nurses
Process data – centre level	Audit	Centre staff

7.1.3 Electronic recording

Study data will be recorded in a number of files for both the administration of the study and collection of participant data.

1. A master index will contain confidential participant contact information and will be the only link between individual participants and the ID number. This will be an Excel spreadsheet.

2. The Forms Tracking index will be identified by ID number only. It will be used to track the data collection forms for each participant for auditing of data collection. It will contain dates of when each form is due, entered and finalised.

3. The Data file will be held and administered in the coordinating site, and will contain all the participant data as contained within the site baseline screening Excel spreadsheet. . This data will then be transferred to the project team for analysis.

7.1.4 Data entry

A designated staff member at each site will enter baseline screening data into an Excel spreadsheet specifically developed for this study. The study site will send the Excel spreadsheet to the coordinating site via secure health service and university email systems.

7.1.5 Data storage

All data collected at each site for each participant will be kept in a participant file (identified by ID number only) which will contain the Case Report Forms, any corrected and amended data, copies of adverse event reports, file notes etc. All data will be stored at each study site and/or the coordinating site in a locked filing cabinet with all identifying information removed, away from the administrative files for the study. Electronic data will be stored on password-protected computers in secure offices at the coordinating university site. Study sites will likewise store baseline screening electronic data generated at their site on password-protected computers in secure offices. All study files will be stored in accordance with Good Clinical Practice guidelines.

At completion of the study, all Case Report Forms will be sent to the sponsor by registered mail, for collation and archiving. All participant files will be reconciled and stored along with all study materials – both hard copy and electronic – consistent with the relevant state regulations regarding the retention and disposal of participant records.

7.2 Quality control

7.2.1 Training procedures:

The following training procedures will be conducted to ensure quality control.

**Person trained**	**Description**	**Assessed by**
All study staff	ICH Good Clinical Practice training	Trial coordinator

Research assistants and trials nurses	Eligibility assessment	Trial coordinator
Research assistants and trials nurses	Consent procedure	Trial coordinator
Research assistants and trials nurses	Data management	Trial coordinator

8.0 References

1. Groenvold M, Petersen MA, Aaronson NK, et al. The development of the EORTC QLQ-C15-PAL: a shortened questionnaire for cancer patients in palliative care. Eur J Cancer. 2006 Jan;42(1):55-64.
2. Maunsell E, Lauzier S, Brunet J, Pelletier S, Osborne RH, Campbell HS. Health-related empowerment in cancer: Validity of scales from the Health Education Impact Questionnaire. Cancer. 2014 Oct 15;120(20):3228-36.
3. Al-Janabi H, Flynn TN, Coast J. Estimation of a Preference-Based Carer Experience Scale. Medical Decision Making. 2011 May/June 2011;31(3):458-68.
4. Woertman W, de Hoop E, Moerbeek M, Zuidema SU, Gerritsen DL, Teerenstra S. Stepped wedge designs could reduce the required sample size in cluster randomized trials. J Clin Epidemiol. 2013 Jul;66(7):752-8.
5. Fitzmaurice G, Laird N, Ware JE. Applied Longitudinal Analysis: John Wiley & Sons; 2004.
6. Briggs AH, Wonderling DE, Mooney CZ. Pulling cost-effectiveness analysis up by its bootstraps: a non-parametric approach to confidence interval estimation. Health Economics. 1997;6(4):327-40.
7. Pope C, Mays N, editors. Qualitative Research in Health Care. London: BMJ 2000.
8. Australian Institute of Health and Welfare & Australasian Association of Cancer Registries. Cancer in Australia: an overview, 2012. Canberra: AIHW2012.
9. Phillips JL, Currow DC. Cancer as a chronic disease. Collegian. 2010;17:47-50.
10. National Breast Cancer Centre & National Cancer Control Initiative (NBCC & NCCI). Clinical Practice Guidelines for the Psychosocial Care of Adults with Cancer. Sydney, Australia: National Breast Cancer Centre; 2003.
11. van den Beuken-van Everdingen MHJ, de Rijke JM, Kessels AG, Schouten HC, van Kleef M, Patijn J. Prevalence of pain in patients with cancer: a systematic review of the past 40 years. Ann Oncol. 2007 September 1, 2007;18(9):1437-49.
12. Deandrea S, Montanari M, Moja L, Apolone G. Prevalence of undertreatment in cancer pain. A review of published literature. Ann Oncol. 2008 Dec;19(12):1985-91.
13. Luckett T, Davidson PM, Green A, Boyle F, Stubbs J, Lovell M. Assessment and management of adult cancer pain: a systematic review and synthesis of recent qualitative studies aimed at developing insights for managing barriers and optimizing facilitators within a comprehensive framework of patient care. Journal of Pain and Symptom Management. 2013;46(2):229-53.
14. Oldenmenger WH, Sillevis Smitt PAE, van Dooren S, Stoter G, van der Rijt CCD. A systematic review on barriers hindering adequate cancer pain management and interventions to reduce them: a critical appraisal. Eur J Cancer. [Review]. 2009 May;45(8):1370-80.
15. Jacobsen R, Sjogren P, Moldrup C, Christrup L. Physician-related barriers to cancer pain management with opioid analgesics: a systematic review. J Opioid Manag. 2007 Jul-Aug;3(4):207-14.
16. Jacobsen R, Moldrup C, Christrup L, Sjogren P. Patient-related barriers to cancer pain management: a systematic exploratory review. Scand J Caring Sci. 2009 Mar;23(1):190-208.
17. Jacobsen R, Liubarskiene Z, Moldrup C, Christrup L, Sjogren P, Samsanaviciene J. Barriers to cancer pain management: a review of empirical research. Medicina (Kaunas, Lithuania). 2009;45:427-33.
18. Fazeny B, Muhm M, Hauser I, et al. Barriers in cancer pain management. Wien Klin Wochenschr. 2000 Nov 24;112(22):978-81.

19. Butow P, Bell M, Goldstein D, et al. Grappling with cultural differences; Communication between oncologists and immigrant cancer patients with and without interpreters. Patient Education and Counseling. 2011;84:398-405.
20. Butow PN, Phillips F, Schweder J, White K, Underhill C, Goldstein D. Psychosocial well-being and supportive care needs of cancer patients living in urban and rural/regional areas: a systematic review. Support Care Cancer. 2012 Jan;20(1):1-22.
21. Dy SM, Asch SM, Naeim A, et al. Evidence-based recommendations for cancer pain management. J Clin Oncol. 2008;26:3879-85.
22. Brink-Huis A, van Achterberg T, Schoonhoven L, Brink-Huis A, van Achterberg T, Schoonhoven L. Pain management: a review of organisation models with integrated processes for the management of pain in adult cancer patients. J Clin Nurs. 2008 Aug;17(15):1986-2000.
23. National Pain Summit Initiative. National Pain Strategy. Melbourne: Faculty of Pain Medicine2010.
24. Flemming K. The use of morphine to treat cancer-related pain: a synthesis of quantitative and qualitative research. Journal of pain and symptom management. 2010;39:139-54.
25. Lovell M, Luckett T, Boyle F, Phillips J, Agar M, Davidson PM. Patient education, coaching and self-management for cancer pain. Journal of Clinical Oncology 2014:doi: 10.1200/JCO.2013.52.4850.
26. Luckett T, Butow PN, King MT. Improving patient outcomes through the routine use of patient-reported data in cancer clinics: future directions. Psychooncology. 2009 Nov;18(11):1129-38.
27. Kotronoulas G, Kearney N, Maguire R, et al. What is the value of the routine use of patient-reported outcome measures toward improvement of patient outcomes, processes of care, and health service outcomes in cancer care? A systematic review of controlled trials. J Clin Oncol. 2014 May 10;32(14):1480-501.
28. Etkind SN, Daveson BA, Kwok W, et al. Capture, Transfer, and Feedback of Patient-Centered Outcomes Data in Palliative Care Populations: Does It Make a Difference? A Systematic Review. J Pain Symptom Manage. 2014 Aug 15.
29. Marie N, Luckett T, Davidson PM, Lovell M, Lal S. Optimal patient education for cancer pain : A systematic review and theory-based meta-analysis. Journal of Supportive Care in Cancer. 2013;21(12):3529-37.
30. Michie S, van Stralen MM, West R. The behaviour change wheel: a new method for characterising and designing behaviour change interventions. Implement Sci. 2011;6:42.
31. Lovell MR, Forder P, Stockler M, et al. A randomised controlled trial of a standardised educational intervention for patients with cancer pain. Journal of Pain and Symptom Management. 2010;40(1):49-59.
32. Lovell M, Agar M, Luckett T, Davidson P, Green A, Clayton J. Australian survey of current practice and guideline use in adult cancer pain assessment and management: Perspectives of palliative care physicians. Journal of Palliative Medicine. 2014;10(2):e99-107. doi: 10.1111/ajco.12040.
33. Luckett T, Davidson PM, Boyle F, et al. Australian survey of current practice and guideline use in adult cancer pain assessment and management: Perspectives of oncologists. Asia Pacific Journal of Clinical Oncology. 2012 doi: 10.1111/ajco.12040.
34. Phillips J, Lovell M, Luckett T, Agar M, Green A, Davidson P. Australian survey of current practice and guideline use in adult cancer pain assessment and management: The community nurse perspective. Collegian. 2014:DOI: http://dx.doi.org/10.1016/j.colegn.2013.11.002.
35. Phillips JL, Hickman L, Heneka N, Shaw T. Assessing specialist palliative care nurses pain assessment capabilities: identifying opportunities to improve patient outcomes. 16th Cancer Nurses Society Australia Conference; 28-29 July 2012; Hobart2012.

36. Palliative Care Outcomes Collaboration. National outcome measures and benchmarks. 2014 [cited 2014 October 21st]; Available from: http://ahsri.uow.edu.au/pcoc/benchmarkmeasures/index.html.
37. Phillips JL, Heneka N, Hickman L, Lam L, Shaw T. Impact of a novel online learning module on specialist palliative care nurses' pain assessment competencies and patients' reports of pain: Results from a quasi-experimental pilot study. Palliat Med. 2014 Mar 31;28(6):521-9.
38. Kerfoot BP, Kearney MC, Connelly D, Ritchey ML. Interactive spaced education to assess and improve knowledge of clinical practice guidelines: a randomized controlled trial. Ann Surg. 2009 May;249(5):744-9.
39. Lovell M, Birch M-R, Luckett T, et al. Pain management can be achieved with commitment and support. NSW State Palliative Care Conference; October; Sydney2014.
40. Lovell M, Birch M-R, Luckett T, et al. Pilot of pain indicator audit tool as part of a complex intervention to improve cancer pain outcomes. World Cancer Congress; December; Melbourne2014.
41. Craig P, Dieppe P, Macintyre S, et al. Developing and evaluating complex interventions: the new Medical Research Council guidance. BMJ. 2008;337:a1655. Also available open-source online at www.mrc.ac.uk/complexinterventionsguidance.
42. Hussey MA, Hughes JP, Hussey MA, Hughes JP. Design and analysis of stepped wedge cluster randomized trials. Contemp Clin Trials. 2007 Feb;28(2):182-91.
43. Australian Bureau of Statistics. 3416.0 - Perspectives on migrants. Canberra: ABS2008.
44. Shaw EK, Howard J, West DR, et al. The role of the champion in primary care change efforts: from the State Networks of Colorado Ambulatory Practices and Partners (SNOCAP). Journal of the American Board of Family Medicine : JABFM. 2012 Sep-Oct;25(5):676-85.
45. National Health and Medical Research Council. National statement on ethical conduct of research. Canberra: NHMRC2007.
46. Hjermstad MJ, Fayers PM, Haugen DF, et al. Studies comparing Numerical Rating Scales, Verbal Rating Scales, and Visual Analogue Scales for assessment of pain intensity in adults: a systematic literature review. J Pain Symptom Manage. 2011 Jun;41(6):1073-93.
47. Cleeland CS, Ryan KM. Pain assessment: global use of the Brief Pain Inventory. Ann Acad Med Singapore. 1994;23(2):129-38.
48. Serlin RC, Mendoza TR, Nakamura Y, Edwards KR, Cleeland CS. When is cancer pain mild, moderate or severe? Grading pain severity by its interference with function. Pain. 1995 May;61(2):277-84.
49. Basch E, Trentacosti AM, Burke LB, et al. Pain palliation measurement in cancer clinical trials: the US Food and Drug Administration perspective. Cancer. 2014 Mar 1;120(5):761-7.
50. Norman GR, Sloan JA, Wyrwich KW. The truly remarkable universality of half a standard deviation: confirmation through another look. Expert Review of Pharmacoeconomics & Outcomes Research. 2004 Oct;4(5):581-5.
51. Aaronson NK, Ahmedzai S, Bergman B, et al. The European Organization for Research and Treatment of Cancer QLQ-C30: a quality-of-life instrument for use in international clinical trials in oncology. Journal of the National Cancer Institute. 1993;85:365-76.
52. Bedard G, Zeng L, Zhang L, et al. Minimal important differences in the EORTC QLQ-C15-PAL to determine meaningful change in palliative advanced cancer patients. Asia Pac J Clin Oncol. 2013 Apr 1.
53. Rowen D, Brazier J, Young T, et al. Deriving a preference-based measure for cancer using the EORTC QLQ-C30. Value in Health. 2011 Jul-Aug;14(5):721-31.
54. Karnofsky DA, Burchenal JH. The Clinical Evaluation of Chemotherapeutic Agents in Cancer. In: MacLeod CM, editor. Evaluation of Chemotherapeutic Agents: Columbia Univ Press; 1949.
55. Abernethy A, Shelby-James T, Fazekas B, Woods D, Currow D. The Australia-modified Karnofsky Performance Status (AKPS) scale: a revised scale for contemporary palliative care

clinical practice. BMC Palliative Care. 2005;4(7):http://www.biomedcentral.com/content/pdf/1472-684X-4-7.pdf.

56. Oken MM, Creech RH, Tormey DC, et al. Toxicity And Response Criteria Of The Eastern Cooperative Oncology Group. Am J Clin Oncol. 1982;5:649-55.

57. de Kock I, Mirhosseini M, Lau F, et al. Conversion of Karnofsky Performance Status (KPS) and Eastern Cooperative Oncology Group Performance Status (ECOG) to Palliative Performance Scale (PPS), and the interchangeability of PPS and KPS in prognostic tools. J Palliat Care. 2013 Autumn;29(3):163-9.

58. R Core Team. R: A language and environment for statistical computing. Vienna, Austria: R Foundation for Statistical Computing; 2012.

59. Yennurajalingam S, Kang JH, Hui D, Kang DH, Kim SH, Bruera E. Clinical response to an outpatient palliative care consultation in patients with advanced cancer and cancer pain. J Pain Symptom Manage. 2012 Sep;44(3):340-50.

60. Norman GR, Sloan JA, Wyrwich KW. Interpretation of changes in health-related quality of life: the remarkable universality of half a standard deviation. Med Care. 2003;41:582-92.

61. Eagar K, Watters P, Currow DC, Aoun SM, Yates P. The Australian Palliative Care Outcomes Collaboration (PCOC)--measuring the quality and outcomes of palliative care on a routine basis. Aust Health Rev. 2010 May;34(2):186-92.

9.0 Appendices

9.1 Appendix 1: Behaviour change functions identified by a systematic review by Michie et al (30) and associated strategies for overcoming barriers to cancer pain assessment and management at the levels of patient, health professional and health system (adapted from (25))

**Behaviour change function and definition**	**Strategies for overcoming barriers to cancer pain assessment and management**		
	Patient level	*Health professional level*	*System level*
**Education** Increasing knowledge or understanding	Information on types of pain, medication and side-effects (especially the low risk of opioid addiction), and when from whom to seek help (OVERCOMING CANCER PAIN BOOKLET)	Information on opioid dosage, conversion and use in patients who are older and/or have renal failure (GUIDELINES)	Data on prevalence of cancer pain (AUDIT)
**Persuasion** Using communication to induce positive or negative feelings or stimulate action	Patient coaching and cognitive behavioural therapy (CBT) aimed reframing pain and promoting sense of control and self-efficacy (PATIENT HELD RESOURCES)	Evidence-base for the need to conduct comprehensive assessment and management strategies; patient advocacy (PATIENT HELD RESOURCES)	Data on hospitalisations and other healthcare costs resulting from cancer pain (ECONOMIC EVALUATION FROM CURRENT STUDY)
**Incentivisation** Creating expectation of reward / **Coercion** Creating expectation of punishment or cost	Resources aimed at elucidating factors exacerbating and alleviating pain and consequences for daily life (PATIENT HELD RESOURCES)	Audit and feedback regarding pain assessment, management and outcomes (AUDIT); national benchmarking (e.g. Palliative Care Outcomes Collaboration (61))	
**Training** Imparting skills	Reliable use of a numerical rating scale to rate pain severity; self-management (e.g. storage and administration of medications) (TRAINING	Undertaking a comprehensive assessment; evaluating risk of opioid abuse; providing patient education (Q STREAM)	Implementation of routine screening for pain (E-SCREENING SYSTEM)
**Environmental restructuring** Changing the physical or social context	Involving carers and family to encourage reporting of pain and provide appropriate support (TRAINING)	GUIDELINES AND PATHWAY	
**Modelling** Providing an example for people to aspire to or imitate	Providing patients with personal stories (e.g. via DVD) of others who have successfully self-managed their pain (OVERCOMING PAIN BOOKLET)	Presentations and grand rounds by expert clinicians (CLINICAL CHAMPIONS)	Centres of excellence
**Enablement** Increasing means/reducing barriers to increase capability or opportunity	Goal setting and question prompt list to support communication between the patient and medical team (PATIENT HELD RESOURCES)		Patient-held record to overcome problems with transfer and coordination of care (PATIENT HELD RESOURCES)

9.2 Appendix 2: Wording at end of screening giving patients opportunity to opt out of being contacted for research purposes

Our service is currently working to improve how we recognise, assess and manage pain. The medical team would like to ask whether you have been experiencing pain and how severe it is. This information is important in helping us to provide you with the best possible care.

To assist in evaluation of this service improvement, a member of the University of Technology Sydney, may contact you, via telephone, to ask about your pain. You will be asked if you are interested in taking part in a phone based research project on pain management.

If you do **NOT** want to be contacted, please tick the following box: ☐

9.3 Appendix 3: Scripts for approaching patients at 1 week

9.3.1 Primary endpoint data

Hello, is that [NAME OF PATIENT]? My name is [NAME OF RESEARCHER]. I am a researcher following up on your visit to [NAME OF CENTRE] last week. At that time, you said that you were experiencing some pain. Is that right? You also gave permission for someone to phone you a week later to see how your pain is, which is what I am doing now. It will take between one and two minutes. Is that OK? We are collecting information about your pain to evaluate the service you've received. With your permission, we will also collect information from your medical record about the type of cancer you have and your age to help make better sense of information about your pain. Giving this information is voluntary, and any information you do provide will be treated confidentially. Can I ask you about your pain now?

(If patient wishes to proceed) Please rate your pain by giving the number between 0 and 10 that best describes your pain at its worst in the last 24 hours where 0 is no pain at all and 10 is pain as bad as you can imagine.

(If patient does not wish to proceed) Of course, that's fine. You don't have to provide any information you don't want to, and your decision not to participate in this research will not affect the care you receive at [NAME OF CENTRE] in any way. We will not contact you again in relation to this project. Thank you for your time. Good bye.

9.3.2 Secondary endpoint data

Hello, is that [NAME OF PATIENT]? My name is [NAME OF RESEARCHER]. I am a researcher following up on your visit to [NAME OF CENTRE] last week. At that time, you said that you were

experiencing some pain. Is that right? You also gave permission for someone to phone you a week later to tell you about some research you can participate in if you choose to, which is what I am doing now. It will take about 15 minutes. Is that OK?

(If patient wishes to proceed)

We are requesting your verbal consent to obtain different kinds of information about your pain and its management.
You should have received a letter through the post together with two information and consent forms. Did you receive these?

(If yes) Can you please go and get one of the information and consent forms so I can talk you through it? We are asking to collect information about your pain, quality of life and use of medications to evaluate the service you've received. You will also have the option of talking to us in more detail about how well you feel your pain has been managed at [NAME OF CENTRE] and how things could have been done better. *(Researcher talks through the information and consent form especially emphasizing that participation is voluntary, the patient can withdraw at any time they choose, and all information will be treated confidentially).*

(*If person chooses to participate in one or more component*) Thank you for agreeing to participate. I need to audio-record your consent so we have a record of what information you have given us permission to use in the project. Are you happy to do that? Please take a moment to read through once again points 1 to 6 on the consent form. Then please read out aloud the sections of point 7 (a, b, and/or c) that refer to information you are happy to provide to the project.

(If no) Can I please post it out to you again? Can I check your address to make sure I've got it right? I can then call you again in a few days' time to go through it with you then.

(If patient does not wish to proceed) Of course, that's fine. You don't have to provide any information you don't want to, and your decision not to participate in this research will not affect the care you receive at [NAME OF CENTRE] in any way. We will not contact you again in relation to this project. Thank you for your time. Good bye.

9.4 Appendix 4: Email circular to centre staff with invitation to participate in anonymous online survey

All clinical and front desk administrative staff are invited to take 1 minute to complete an anonymous online survey about the time they have spent on screening, assessment and management of pain in people with cancer during their most recent completed work day. This information will contribute to analysis of cost-effectiveness for the Stop Cancer PAIN Trial currently underway at [centre's name]. The survey link is [survey link]. For more information, please contact [contact details].

9.5 Appendix 5: Anonymous online survey questions to staff

Please identify your role at [centre's name] and indicate the number of hours and minutes you have spent on screening, assessing and managing pain in people with cancer during your most recent completed working day.

The information you provide is anonymous and will be used in analysis of cost-effectiveness for the Stop Cancer PAIN Trial currently underway at [centre's name]. Information about your role is needed to estimate the cost associated with your time. Completing the survey will be taken as consent to use the information in this way.

Your role at [centre's name]	Front desk administrative / medical / nursing / allied health
Screening pain in people with cancer	_ _ hours _ _ minutes
Assessing pain in people with cancer	_ _ hours _ _ minutes
Managing pain in people with cancer	_ _ hours _ _ minutes

Thank you

9.6 Appendix 6: Email circular inviting participation in staff focus groups

All clinical staff and front desk administrative staff at [centre's name] are invited to give their feedback on strategies implemented as part of the Stop Cancer PAIN Trial. Staff are invited to take part in focus groups aimed at understanding what has worked well or could be improved in rolling out the strategies to improve assessment and management of cancer pain. Focus groups will take about 30 minutes and will be held at [centre's name] through the week beginning [date]. Please RSVP to [email address] if you would like to participate or are interested in finding out more information. Staff are welcome to participate in a one-to-one interview as an alternative if they prefer.

9.7 Appendix 7: Survey tools

What number describes your worst/average pain over the past 24 hours, where zero is no pain and ten is worst pain you can imagine.

0	1	2	3	4	5	6	7	8	9	10
No pain										worst pain you can imagine

EORTC QLQ-C15-PAL (version 1)

We are interested in some things about you and your health. Please answer all of the questions yourself by circling the number that best applies to you. There are no "right" or "wrong" answers. The information that you provide will remain strictly confidential.

Please fill in your initials: |_|_|_|_|
Your birthdate (Day, Month, Year): |_|_|_|_|_|_|_|_|
Today's date (Day, Month, Year): |_|_|_|_|_|_|_|_|

	Not at All	A Little	Quite a Bit	Very Much
1. Do you have any trouble taking a <u>short</u> walk outside of the house?	1	2	3	4
2. Do you need to stay in bed or a chair during the day?	1	2	3	4
3. Do you need help with eating, dressing, washing yourself or using the toilet?	1	2	3	4

**During the past week:**	Not at All	A Little	Quite a Bit	Very Much
4. Were you short of breath?	1	2	3	4
5. Have you had pain?	1	2	3	4
6. Have you had trouble sleeping?	1	2	3	4
7. Have you felt weak?	1	2	3	4
8. Have you lacked appetite?	1	2	3	4
9. Have you felt nauseated?	1	2	3	4

Specimen

**During the past week:**	**Not at All**	**A Little**	**Quite a Bit**	**Very Much**
10. Have you been constipated?	1	2	3	4
11. Were you tired?	1	2	3	4
12. Did pain interfere with your daily activities?	1	2	3	4
13. Did you feel tense?	1	2	3	4
14. Did you feel depressed?	1	2	3	4

For the following question please circle the number between 1 and 7 that best applies to you

15. How would you rate your overall quality of life during the past week?

1	2	3	4	5	6	7
Very poor						Excellent

Specimen

Five additional items from the EORTC QLQ-C30 that will be added to the QLQ-C15-PAL to enable estimation of QALYs for cost-utility analysis

	Not at All	A Little	Quite a Bit	Very Much
Do you have any trouble taking a long walk?	1	2	3	4
Were you limited in doing either your work or other daily activities?	1	2	3	4
Have you had diarrhoea?	1	2	3	4
Has your physical condition or medical treatment interfered with your family life?	1	2	3	4
Has your physical condition or medical treatment interfered with your social activities?	1	2	3	4

Health Education Impact Questionnaire

1 strongly disagree; 2, disagree; 3, agree; 4, strongly agree

Social integration and support

1. If I need help, I have plenty of people I can rely on
2. I have enough friends who help me cope with my health
3. When I feel ill, my family and carers really understand
4. Overall, I feel well looked after by friends or family
5. I get enough chances to talk about my health

Health service navigation

6. I have very positive relationships with my health care
7. I communicate very confidently with my doctors about
8. I confidently give health care professionals the information
9. I get my needs met from available health care resources
10. I work in a team with my doctors and other health care

Constructive attitudes and approaches

11. I try not to let my health problems stop me from enjoying life
12. My health problems do not ruin my life
13. I feel I have a very good life even when I have health problems
14. I do not let my health problems control my life
15. If others can cope with problems like mine, I can too

Skill and technique acquisition

16. I have effective ways to prevent my health problems
17. I have a very good idea of how to manage my health problems
18. When I have health problems, I have skills that help me coped
19. I have a good understanding of equipment that could make my life easier

Carer Experience Scale

PLEASE TICK ONE BOX FOR EACH GROUP to indicate which statement best describes your current caring situation.

1.Activities outside caring *(Socialising, physical activity and spending time on hobbies, leisure or study)*

You can do most of the other things you want to do outside caring .... ☐1
You can do some of the other things you want to do outside caring .... ☐2
You can do few of the other things you want to do outside caring .... ☐3

2. Support from family and friends *(Personal help in caring and/or emotional support from family, friends, neighbours or work colleagues)*

You get a lot of support from family and friends .... ☐1
You get some support from family and friends .... ☐2
You get little support from family and friends .... ☐3

3. Assistance from organisations and the Government *(Help from public, private or voluntary groups in terms of benefits, respite and practical information)*

You get a lot of assistance from organisations and the Government .... ☐1
You get some assistance from organisations and the Government .... ☐2
You get little assistance from organisations and the Government .... ☐3

4. Fulfilment from caring *(Positive feelings from providing care, which may come from: making the person you care for happy, maintaining their dignity, being appreciated, fulfilling your responsibility, gaining new skills or contributing to the care of the person you look after)*

You mostly find caring fulfilling .... ☐1
You sometimes find caring fulfilling .... ☐2
You rarely find caring fulfilling .... ☐3

5. Control over the caring *(Your ability to influence the overall care of the person you look after)*

You are in control of most aspects of the caring .... ☐1
You are in control of some aspects of the caring .... ☐2
You are in control of few aspects of the caring .... ☐3

6. Getting on with the person you care for *(Being able to talk with the person you look after, and discuss things without arguing)*

You mostly get on with the person you care for .... ☐1
You sometimes get on with the person you care for .... ☐2
You rarely get on with the person you care for .... ☐3

© Hareth Al-Janabi, Joanna Coast & Terry Flynn

***Australia - Modified Karnofsky Performance Status**

- ○ 100=Normal; no complaints; no evidence of disease.
- ○ 90 = Able to carry on normal activity; minor signs or symptoms.
- ○ 80 = Normal activity with effort; some signs or symptoms of disease.
- ○ 70 = Cares for self; unable to carry on normal activity or to do active work.
- ○ 60 = Requires occasional assistance but is able to care for most of his needs.
- ○ 50 = Requires considerable assistance and frequent medical care
- ○ 40 = In bed more than 50% of the time.
- ○ 30 = Almost completely bedfast.
- ○ 20 = Totally bedfast and requiring extensive nursing care by professionals and/or family.
- ○ 10 = Comatose or barely rousable.
- ○ 0 = Dead.
- ○ Not collected

ECOG PERFORMANCE STATUS	
**Grade**	**ECOG**
0	Fully active, able to carry on all pre-disease performance without restriction
1	Restricted in physically strenuous activity but ambulatory and able to carry out work of a light or sedentary nature, e.g., light house work, office work
2	Ambulatory and capable of all selfcare but unable to carry out any work activities. Up and about more than 50% of waking hours
3	Capable of only limited selfcare, confined to bed or chair more than 50% of waking hours
4	Completely disabled. Cannot carry on any selfcare. Totally confined to bed or chair
5	Dead

9.8 Appendix 8: Interview guide for patients and caregivers

1. How well has pain been managed in your case? *(Note – if interviewing a carer, substitute 'your' with 'of the person you care for' in each case)*
2. What (if anything) has worked well in managing your pain?
3. What (if anything) has not worked well in managing your pain?
4. How could pain management have been improved for you?
5. Which services and health professionals have been involved in managing your pain?
6. How well has pain management been coordinated between these different services and health professionals?
7. Have you been asked to rate your pain on a 0 to 10 scale or some other way?
8. How has pain been assessed?
9. What (if any) pain medications have you been prescribed to take regularly?
10. What (if any) plan have you been given for what to do if the regular medication isn't working as well as it should?
11. What (if any) help have you been given with potential side-effects?
12. What (if any) other advice or assistance with managing your pain, apart from taking medications?
13. What (if any) information and training have you been given?
14. What (if any) advice have you been given regarding people you can contact for help?
15. Can you please show me any resources you've been given to help manage your pain?
16. What (if anything) has worked well among these resources?
17. What (if anything) has not worked well among these resources?
18. How could the resources be improved do you think?
19. Is there anything else you would like to add that I haven't asked about?

9.9 Appendix 9: Focus group/interview guide for centre staff

1. Since this project started, what (if anything) has changed at your centre with regard to the screening, assessment, management of cancer pain?
2. What (if anything) has worked well about the strategies introduced to help your centre better screen, assess and manage cancer pain?
3. What (if anything) has not worked well?
4. How could the strategies have been improved?
5. What (if any) other strategies might help?
6. Have the strategies influenced how you work as a team?
7. Have there been any influences on other aspects of patient care?
8. Is there anything else you would like to add that I haven't asked about?

9.10 Appendix 10: Incident reporting template

**Date of incident**		**Source of report**	
**Date of reporting**		**Name of Stop Pain staff who received report**	
**Method of reporting**	Formal ☐	Conversation ☐	Email ☐
	Telephone ☐	Meeting ☐	Other: ___________
**Incident:**			
<u>Detail:</u>			
**Action:**			
**Date this template completed**			
**Signature**			
