# Supplementary material for: Effect of Cancer Pain Guideline Implementation on Pain Outcomes Among Adult Outpatients With Cancer-Related Pain: A Stepped Wedge Cluster Randomized Trial
Source: JAMA Netw Open. 2022 Feb 21;5(2):e220060. doi: 10.1001/jamanetworkopen.2022.0060 (PMC8861847; doi:10.1001/jamanetworkopen.2022.0060)
Supplement: Supplement 2. — Data Sharing Statement [file jamanetwopen-e220060-s002.pdf]

Data Sharing Statement

Lovell. Effect of Cancer Pain Guideline Implementation on Pain Outcomes Among Adult Outpatients With Cancer-Related Pain. *JAMA Netw Open*. Published February 21, 2022. doi:10.1001/jamanetworkopen.2022.0060

Data
Data available: No

Additional Information
Explanation for why data not available: Patient consent was not obtained to share data
